# Immunomodulatory effects of inulin and its intestinal metabolites

**DOI:** 10.3389/fimmu.2023.1224092

**Published:** 2023-08-10

**Authors:** Wei Sheng, Guang Ji, Li Zhang

**Affiliations:** Institute of Digestive Diseases, Longhua Hospital, Shanghai University of Traditional Chinese Medicine, Shanghai, China

**Keywords:** inulin, short-chain fatty acids, intestinal immunity, intestinal microbiota, intestinal epithelial cells, intestinal immune cells

## Abstract

“Dietary fiber” (DF) refers to a type of carbohydrate that cannot be digested fully. DF is not an essential nutrient, but it plays an important part in enhancing digestive capacity and maintaining intestinal health. Therefore, DF supplementation in the daily diet is highly recommended. Inulin is a soluble DF, and commonly added to foods. Recently, several studies have found that dietary supplementation of inulin can improve metabolic function and regulate intestinal immunity. Inulin is fermented in the colon by the gut microbiota and a series of metabolites is generated. Among these metabolites, short-chain fatty acids provide energy to intestinal epithelial cells and participate in regulating the differentiation of immune cells. Inulin and its intestinal metabolites contribute to host immunity. This review summarizes the effect of inulin and its metabolites on intestinal immunity, and the underlying mechanisms of inulin in preventing diseases such as type 2 diabetes mellitus, inflammatory bowel disease, chronic kidney disease, and certain cancer types.

## Introduction

“Dietary fiber” (DF) is defined as carbohydrate polymers containing ≥ 10 monomeric units that resist digestion by endogenous enzymes in the small intestine. DF includes edible carbohydrate polymers that exist naturally in food, and carbohydrate polymers that are synthesized by physical, chemical, or enzymatic methods (1). DF can be divided into “soluble DF” (SDF) and “insoluble DF” (IDF) according to solubility, and “partially fermentable fiber” and “completely fermentable fiber” by its fermentability (2). The microfibrils formed by the inter- and intra-molecular hydrogen bonds can hinder the degradation and utilization of partially fermentable fiber, which prevent its fermentation in the intestine (3). The health benefits of DF are manifested mainly by altering gut microbiota composition and microbial metabolites.

Inulin is one kind of SDF. It is a type of fructan derived mainly from plants such as chicory, ginger, garlic, onion, and asparagus. “Inulin” is a generic term covering all β- (2, 1) linear fructans, and inulin-type fructans must have β-(2,1) linkages, which give inulin unique structural and physiological properties, making it resistant to enzymatic hydrolysis by human saliva and small intestinal digestive enzymes (4). Most inulin-type fructans have an average degree of polymerization of 10-12 and a chain length of 2-60 units of molecular distribution (5). Oligofructose can be hydrolyzed from inulin by inulinase into a chain length from 2 to 10. Therefore, the sugar chain of inulin is longer compared with that of oligofructose, resulting in slower fermentation and gas production. Inulin has been used widely as a prebiotic, fat substitute, sugar substitute, texture modifier, and in the development of functional foods (6). The US Department of Agriculture recommends consuming 25-36 g of fiber daily (or 14 g for every 1000 calories per day) (7). In 2003, the US Food and Drug Administration (FDA) categorized inulin as “generally recognized as safe”. The daily effective intake is 5 g, and the recommended maximum daily intake is 15-20 g (8). Nausea, bloating, and flatulence are the most common adverse effects of taking inulin. Inulin consumption under 40 g per day in healthy adults is safe. However, inulin can cause serious side effects in patients with inflammatory bowel disease (IBD) or allergies.

The intestine is the front-line of the body’s defense, and is exposed to many pathogens and bacteria. As the largest immune organ of the body, the intestinal immune system (also known as the mucosal immune system) is composed mainly of intestinal epithelial cells (IECs), lamina propria-lymphocytes, intraepithelial lymphocytes, and the Peyer’s patch. An inulin-rich diet has been reported to improve the function of the intestinal barrier and modulate the immune system (9).

The aim of this review is to focus on the immunomodulatory effects of inulin and its intestinal metabolites. In this way, we hope to provide a comprehensive overview of the role of inulin and its metabolites in different diseases.

## Intestinal metabolites of inulin

As mentioned above, the unique β-configuration in the monomeric isomer C2 of fructose prevents inulin-type fructose from being hydrolyzed by digestive enzymes (including α-glucosidase, maltosidase, and sucrase) (10). Upon the fermentation of intestinal bacteria, inulin produces lactate and short-chain fatty acids (SCFAs), including acetate, butyrate and propionate, as well as gases that are excreted from the body eventually (11–15) (**Table 1**). Notably, lactate does not usually accumulate in the healthy gut because microbes can convert it further to propionate, butyrate, or acetate (25). The degree of fermentation of DF is closely correlated with its composition. SDFs such as inulin are usually more fermentable than IDFs and produce more gas and SCFAs (16, 26). In addition, the fermentation properties of inulin are related to the length of its sugar chain; short-chain inulin is more soluble in water than long-chain inulin. Muthyala and colleagues reported changes in fecal SCFA levels in mice of different ages after inulin ingestion. They found butyric acid to be the main metabolite in middle-aged mice, whereas the fecal level of propionic acid showed an age-dependent decrease. Those evidences suggest that age is an important factor influencing inulin metabolism by the intestinal microbiota (27).

**Table 1:** Metabolites induced by inulin fermentation.

Interventions	Duration	Models or subjects	Metabolites with significantly upregulated expression	References
Inulin (10 g/L)	24 h	Fresh stool samples from 9 healthy humans (*ex vivo* system)	Acetate, propionate, and butyrate	(14)
FOS (12 g/d)-enriched inulin supplementation	0, 12, 24, and 48 h	Fecal cultures from pigs (*in vitro* fecal fermentation)	Succinate, lactate, propionate and butyrate	(16)
Inulin (24 g) plus glucose (75 g)/water (300 mL)	0-6 h	25 adults with BMI of 20-35 kg/m^2^	Propionate and butyrate	(15)
Inulin (24 g) plus high-fructose corn syrup (56 g)/drinks (400 mL)	4-6 h	12 healthy humans	Serum acetate, propionate, and butyrate	(17)
U-^13^C-inulin (0.5 g)/inulin (24 g) in a high-fat milkshake	7 h	14 healthy, overweight to obese men	Plasma propionate, butyrate, acetate	(18)
Inulin-type fructans	6 weeks	25 patients with type 2 diabetes mellitus	Significantly increased fecal concentrations of total short-chain fatty acids, acetic acid and propionic acid	(19, 20)
Water with 20% sucrose and 5% inulin (*w/w*)	6 weeks	Male Sprague–Dawley rats (6 weeks)	Propionate and butyrate; fecal contents of indole-3-acetic acid and kynurenic acid	(21)
Basal diet containing 0.5% inulin	21 days	20 growing-pigs	Acetate and butyrate concentrations in cecum	(22)
Control diet with 20% inulin	3 weeks	BALB/c mice (6–8 weeks)	Fecal acetate, propionate and butyrate	(23)
High-fat/high-sucrose diet containing inulin (7.5% kcal)	12 weeks	Male C57BL/6J mice (8 weeks)	Acetic acid in jejunum; succinic acid, acetic acid and propionic acid in the rectal feces and portal vein serum	(24)

More interestingly, inulin and gut microbiota are mutually interacted. Gut bacteria ferment inulin to produce the corresponding metabolites. Likewise, the gut microbiome responds to inulin treatment and exhibits significant structural alterations. Inulin treatment promotes the growth of certain beneficial bacteria as well as bacteria that promote the production of SCFAs, such as *Bifidobacterium* spp (28). SCFAs can act locally in the intestine and be used as energy sources by intestinal mucosal cells to promote barrier function and maintain mucosal immunity, and provide energy substrate for colonic cells (29, 30). SCFAs can also enter the circulation through the hepatic portal vein and act as signaling molecules, thereby regulating systemic immune function (31, 32). The G protein-coupled receptors (GPCRs) GPR43 and GPR41 were the first GPCRs to be identified as activated by SCFAs, and were subsequently renamed as the specific free fatty acid receptors (FFARs) FFAR2 and FFAR3, respectively. Recently, three additional GPCRs, GPR109A, Olfr78 and Olfr558, were identified as receptors for SCFAs (33). SCFAs are involved in the regulation of inflammatory responses because they interact with these receptors expressed on innate immune cells (34). Notably, FFAR2/3 has been found to be expressed mainly in enteroendocrine cells and immune cells. Expression of FFARs on colonic regulatory T (T_reg_) cells has been shown to be significantly higher than that on other tissues (34, 35), suggesting a potential role of SCFAs in maintaining intestinal immune homeostasis.

## Effects of inulin and its metabolites on intestinal microbiota

The human intestinal microbiota is divided into four major phyla covering more than 90% of the total bacterial population, i.e., *Firmicutes*, *Bacteroidetes*, *Actinobacteria*, *Proteobacteria* and other minor phyla, including Warty microbes and *Clostridium* (36). The phylum *Firmicutes* and *Bacteroidetes* are the two most abundant microbial phyla in the human intestinal microbiota. The *Firmicutes* are low GC Gram-positive bacteria, and *Clostridium* spp. and *Lactobacillus* spp. are the dominant component, while the leading members of *Bacteroidetes* are *Bacillus* spp. and *Prevotella* spp. Increased ratio of Phylum *Firmicutes*/*Bacteroidetes* is usually considered to be associated with obesity-associated dysbiosis (37, 38). Inulin intake has been reported to significantly reduce the ratio of *Firmicutes* and *Bacteroidetes*, as well as levels of several bacteria associated with a pro-inflammatory state (27). Bastard and colleagues also found that changes in intestinal microbiota after inulin supplementation decreased the relative abundance of *Bacteroidetes*, and increased levels of *Bifidobacterium* spp., *Anaerostipes* spp., *Enterococcus faecalis*, and *Lactobacillus* spp (39).. Inulin also promotes an increase in the abundance of bacteria of the genera *Phascolarctobacterium*, *Blautia*, *Akkermansia*, *Ruminococcus*, and the family *Lachnospiraceae*, which are also responsible for SCFAs production (21, 40, 41). We have summarized some major changes in intestinal microbiota after at least 4 weeks or even 3 months of inulin supplementation in different models or individuals, as shown in **Table 2**.

**Table 2:** Examples of microbiota modulation after inulin ingestion.

Treatment	Duration	Models or subjects	Altered gut microbiota level		Reference
			Up-regulation	Down-regulation	
Diet of inulin (2.5 g or 5 g/100 g	4 weeks	*ob*/*ob* mice	*Ruminococcaceae*, *Lachnospiraceae*, *Bacteroides*, and *Bifidobacterium*	–	(42)
Inulin (16 g/d)	3 months	Obese patients	*Bifidobacterium*, *Catenibacterium*, *Erysipelotrichaceae incertae sedis*, *Escherichia/Shigella*, *Lact-bacillus*, and *Dorea*	*Desulfovibrio, Roseburia*, *Butyricimonas*, *Clostridium* cluster XIVa, and *Clostridium sensu stricto*	(43)
An inulin-containing semi- purified, irradiated regular diet	6 weeks	Male C57BL/6J mice	*Akkermansia*, *Roseburia*, *Bacteroides*	*Lactococcus*, *Ruminiclostridium_9*, *Ruminococcaceae* and *Streptococcaceae*	(27)
Vilof™ soluble dietary fiber powder (3 g/kg bodyweight/d) containing 91% inulin-type fructans	12 weeks	Model of diabetes mellitus in rat	*Lactobacillus*, Lachnospiraceae, *Bacteroides*, and *Phascolarctobacterium*	*Desulfovibrio*	(40)
Water with 20% sucrose and 5% inulin (*w/w*)	6 weeks	Male Sprague-Dawley rats	*Bifidobacterium*, *Actinobacteria*, *Blautia* and *Phascolarctobacterium*	*Proteobacteria*	(21)
Lieber–DeCarli liquid diets containing inulin (0.5 g/L)	6 weeks	Female C57BL/6J mice	*Allobaculum*, *Lactobacillus* and *Lactococcus*	*Parasutterella*	(44)
Inulin-propionate ester (20 g/d)	42 days	Overweight or obese adults not suffering from diabetes mellitus	*Actinobacteria*	*Clostridia*	(45)

In addition to promoting SCFAs production, *Bifidobacteria* spp. are also considered to be probiotics that inhibit the proliferation of pathogenic bacteria. Dietary inulin supplementation increases the relative abundance of *Bifidobacteria* spp. and consequently brings a series of beneficial alterations defined as “bifidogenic effects” (28). Thus, inulin use inhibits harmful bacteria or opportunistic pathogens by promoting the proliferation of beneficial bacteria. In addition, pathogenic bacteria tend to colonize in the intestine with an alkaline environment. Inulin lowers intestinal pH after enterobacterial fermentation, which also contributes to the inhibition of pathogenic bacteria (26). Furthermore, treatment with inulin has been shown to significantly reduce the abundance of lipopolysaccharide (LPS)-producing *Desulfovibrio* spp. in rats and obese patients (40, 43), which may protect the intestinal barrier from endotoxin damage.

Inulin has been reported to be beneficial for a series of diseases through modulating intestinal microbiota. A reduced abundance of *F. prausnitzii* has been observed in patients with nonalcoholic fatty liver disease (NAFLD). Inulin can provide carbon sources for the transporter of the fructose phosphotransferase system. This action enhances the fructose-absorption activity of *F. prausnitzii* and increases the abundance of *F. prausnitzii* in the gut (46). In a model of alcoholic fatty liver disease, chronic exposure to alcohol resulted in decreased abundance of the genera *Allobaculum*, *Lactobacillus*, and *Lactococcus*, but increased abundance of *Parasutterella* species. Inulin could reverse these alterations and reduce the number of macrophages (44). In addition, dietary supplementation with inulin has been found to restore the diversity of intestinal microbiota in a mouse model of obesity based on high-fat-diet (HFD) consumption (47).

## Effects of inulin and its metabolites on IECs

Reduced intestinal mucosal tolerance promotes immune-mediated inflammatory diseases. The most important part of the intestinal mucosal barrier is the intestinal mucosal mechanical barrier. The latter is a defense layer composed of intestinal mucosal epithelial cells and tight junctions (TJs) that protects against pathogens (48). IECs are differentiated from intestinal stem cells and can be divided broadly into “absorptive enterocytes” and “secretory enterocytes” (goblet cells that secrete mucus, Paneth cells that secrete antimicrobial peptides and immunomodulatory proteins, and enteroendocrine cells that secrete hormone) (49, 50).

Inulin-type fructans extracted from different plants have been shown to have direct immunomodulatory effects on IECs. For example, inulin-type fructans from *Platycodon grandiflorus* have been shown to stimulate transcription of the anti-inflammatory factors interleukin (IL)-4 and IL-10 in a dose-dependent manner in a porcine jejunum epithelial cell line (IPEC-J2) (51). The inulin fractions from *Codonopsis pilosula* and *Codonopsis tangshen* are natural sources of potential antioxidants, which can increase intestinal levels of glutathione peroxidase, superoxide dismutase and catalase, but reduce the levels of malondialdehyde and lactate dehydrogenaseto enhance the antioxidant defense of IECs (52). Moreover, *Ruminococcus bromii*-producing butyrate is a major source of energy for colonocytes, which contributes to enterocyte proliferation (53, 54).

In addition to promoting the function of IECs, inulin can promote intestinal barrier function by regulating TJ proteins. Dietary supplementation with inulin can restore the integrity and function of the intestinal barrier by promoting the expression of zonula occludens (ZO)-1, claudin-1 and occludin (21). Chen et al. found that long-chain inulin-type fructans enhanced expression of the intestinal-barrier TJ proteins occludin and claudin-2, antimicrobial peptides β-defensin-1, cathelicidin-related antimicrobial peptide, and SCFAs production (55). In another animal experiment, inulin supplementation increased villus height and ZO-1 expression, reduced secretion of IL-6 and tumor necrosis factor (TNF)-α, and increased IECs apoptosis in the ileum and cecum (22). Mucin 2 (Muc2) is the main component of mucus. Muc2 can constrain the immunogenicity of antigens by forming a non-specific physical barrier. A previous study showed that Muc2 can be ingested by dendritic cells (DCs), and reduce the number of inflammatory DCs by inhibiting gene transcription through nuclear factor-kappa B (NF-κB). Therefore, Muc2 can increase the tolerance of the intestine (56). Inulin has also been found to promote the secretion of Muc2 and secretory immunoglobulin A (sIgA) in the ileum (57). sIgA is involved in important mucosal immune functions against external antigens on human mucosal surfaces. Thus, inulin intake facilitates the protection of IECs from luminal bacteria and food antigens, and enhances intestinal homeostasis and tolerance to prevent inflammation. In addition, the effect of inulin on host defense in Paneth cells may be mediated (at least in part) by SCFAs produced by inulin fermentation. Supplementation with inulin has been shown to induce expression of α-defensin and matrix metalloproteinase (MMP)-7 from Paneth cells in an obese mouse model. Moreover, organoid culture of small intestinal crypts revealed that the fermentation products of inulin induced α-defensin expression from Paneth cells (58). Butyrate has also been found to enhance the intestinal barrier by activating adenosine monophosphate-activated protein kinase to promote TJ assembly in monolayers of Caco-2 cells (59).

In general, consumption of an inulin-containing diet is beneficial for intestinal health. However, some studies have reported contradictory evidences. One study showed that a moderate dose of inulin (50 mg per mouse) was beneficial against food allergy, whereas high-dose inulin supplementation (80 mg per mouse) increased serum levels of allergic inflammation-related factors and an intestinal inflammatory response. Further profiling indicated that the altered intestinal TJ proteins and T cell homeostasis seen in hyperinulin-treated mice might be related to the high production of SCFAs by bacteria of the family *Ruminococcaceae* and *Bifidobacterium* spp (60). In addition, long-term intake of inulin also exacerbated intestinal damage and inflammatory responses in the progeny of rats in a dextran sodium sulfate (DSS)-induced colitis model (61).

## Effects of inulin and its metabolites on intestinal immune cells

**Figure 1:**
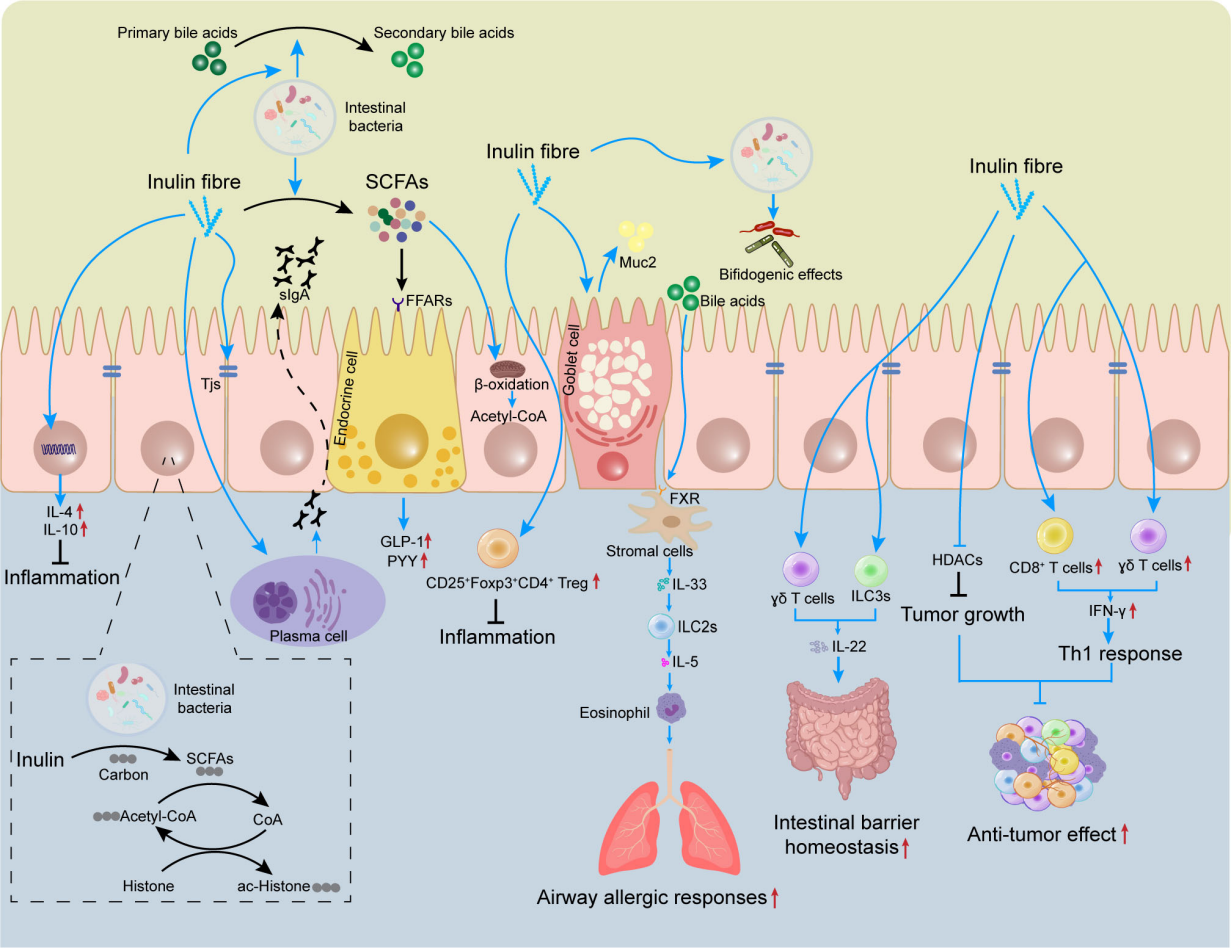
Effects of inulin on the mucosal immune system. The gut contains many immune cells. Inulin can regulate the differentiation and proliferation of these immune cells (e.g., T_reg_) to limit intestinal inflammation. Inulin promotes expression of TJ proteins and induces secretion of sIgA and Muc2 by plasma cells and goblet cells, which helps to maintain intestinal-barrier homeostasis. Inulin promotes IL-22 secretion by γδ T cells and ILC3s, which also helps to improve the intestinal barrier. However, inulin increases the circulating level of bile acids and triggers ILC2s to induce eosinophils, thereby exacerbating airway allergic responses. Inulin provides carbon sources for histone acetylation, regulates epigenetics, and inhibits tumor growth. In tumors, inulin can also promote the infiltration of CD8^+^ T cells and γδ T cells to enhance the anti-tumor effect. SCFAs, short-chain fatty acids; sIgA, secretory immunoglobulin A; FFARs, free fatty acid receptors; Muc2, mucin 2; TJ, tight junction; GLP-1, glucagon-like peptide 1; PYY, peptide YY, FXR, farnesoid X receptor; HDAC, histone deacetylase; ILC, innate lymphoid cell.

Many types of immune cell, such as T cells, innate lymphoid cells (ILCs), and macrophages, are present in the lamina propria of the intestine. Mucus and antimicrobial peptides secreted by goblet cells, as well as immunomodulatory proteins secreted by Paneth cells, can help to prevent the adhesion of pathogenic bacteria and viruses in the intestinal lumen (62, 63). Several studies have shown that the role of inulin in regulation of immune cell activation and cytokine secretion is largely dependent on its intestinal metabolites, such as SCFAs. The latter can act directly on host T cells by reprogramming their metabolic activity and epigenetic status to control the differentiation of effector T (T_eff_) cells and T_reg_ cells (64). More importantly, SCFAs can also enter the circulation and regulate the function of immune cells in other tissues (65). The effects of inulin and its metabolites on immune cells is summarized in **Figure 1**.

### T_eff_ and T_reg_ cells

T cells are critical mediators of adaptive immunity. When T cells recognize pathogens through T-cell receptors, together with costimulatory signals provided by antigen-presenting cells, T cells expand clonally and traffic to tissues, thereby triggering an adaptive immune response. However, an excessive immune response usually leads to severe tissue damage. In contrast, T_reg_ cells can limit the immune response from T_eff_ cells to avoid overwhelming inflammatory responses, a process known as “immune tolerance” (66). Several recent studies have shown SCFAs to be critical factors in balancing adaptive immunity and immune tolerance (28, 67). SCFAs produced by inulin fermentation maintain immune homeostasis by suppressing excessive innate responses and stimulating specific adaptive immunity.

The metabolic and functional changes of cluster of differentiation (CD)8^+^ T cells are partially mediated by inulin and SCFAs. Inulin treatment promotes the infiltration CD8^+^ T cells in tumors of several mouse models, and induces a shift to a pro-inflammatory tumor microenvironment (68–70). Furthermore, SCFAs (e.g., butyrate) can regulate the metabolism of CD8^+^ T cells by acting on FFAR3, thereby ensuring rapid and sustained activation of T_eff_ cells during viral infections (28). In contrast, the mechanisms by which inulin and SCFAs limit autoimmune responses by regulating T_reg_ cells differentiation are more complex. Butyrate promotes production of extra-thymic T_reg_ cells in an intronic enhancer CNS1-dependent manner if administered systemically, but increases only intracolonic T_reg_ cells production if administered locally via an enema (71). Conversely, acetate and propionate promote the accumulation of intracolonic T_reg_ cells in an FFAR2-dependent manner (66).

IL-10 is a key cytokine for T_reg_ cells to exert anti-inflammatory effects. IL-10 is secreted by T_reg_ subsets that express the transcription factor forkhead box P3 (*Foxp3*). Thus, T_reg_ cells expressing *Foxp3* are crucial in limiting intestinal inflammatory responses (34). An independent study showed that supplementation with long-chain inulin-type fructans promoted the proliferation of CD25^+^ Foxp3^+^ CD4^+^ T_reg_ cells and reduced the number of IL17A^+^ CD4^+^ T-helper (Th)17 cells, thereby modulating T cell responses and suppressing intestinal inflammatory responses (55). In addition, metabolites of inulin (e.g., propionate) regulate the proliferation and differentiation of CD25^+^ Foxp3^+^ T_reg_ cells (66, 72). Propionate also improves angiotensin II-induced inflammatory responses by modulating T_reg_ cells (73).

Histone acetyltransferase (HAT) and histone deacetylase (HDAC) are important for regulating gene expression. Usually, a high acetylation level indicates active transcriptional activity. A low acetylation level is associated with transcriptional repression. In the intestine, if HDAC is overexpressed, the balance of gene expression is disrupted and cell proliferation is abnormal, which eventually leads to tumorigenesis (74). In contrast, the gut microbiota can inhibit HDAC activity by fermenting inulin into SCFAs, thereby regulating the acetylation level of histones and affecting epigenetic changes in immune cells (75, 76). An independent study showed that inulin dietary treatment inhibited HDACs activity (including HDAC2 and HDAC8), and induced protective epigenetic changes in mouse mammary tumor cells (77). In another animal experiment, consumption of an inulin diet increased the level of SCFAs (especially butyrate), which enhanced the host antimicrobial program by inhibiting HDAC3 (23). Similarly, Fernández et al. found that administration of inulin-rich products reduced the number of colon polyps in two animal models of colorectal cancer (CRC), which may be related to HDACs regulation (78). Furthermore, a study using isotope tracing revealed inulin-derived SCFAs to provide carbon sources for histone acetylation (76).Notably, SCFAs are also involved in regulating epigenetic changes and energy metabolism of B cells by suppressing the activity of HDACs (79–82).

In particular, SCFAs contribute significantly in the fight against intestinal inflammation by promoting T_reg_ cells differentiation through the inhibition of HDACs. Butyrate has also been found to be a potent inhibitor of HDACs (83). Furusawa and colleagues found that butyrate produced by obligate anaerobic bacteria improved colitis by promoting histone H3 acetylation in the promoter and conserved non-coding sequence regions of the *Foxp3* locus, thereby supporting differentiation of T_reg_ cells and enhancing intestinal immune tolerance (84). In addition to directly promoting the differentiation of CD4^+^ T-cell precursors into T_reg_ cells, butyrate and propionate can induce the differentiation of extrathymic T_reg_ cells and reduce expression of pro-inflammatory cytokines within DCs by inhibiting HDACs activity (71). Propionate enhances histone acetylation in colonic T_reg_ cells, drives the proliferation and differentiation of T_reg_ cells, and enhances T_reg_ cell-mediated inhibition of colitis. These effects of propionate appear to be dependent on activation of FFAR2 (66). Nevertheless, whether the inhibitory effect of SCFAs on HDACs is dependent on expression of FFAR2 and FFAR3 is controversial, because SCFAs seem to enter cells directly through membrane transport proteins on the cell surface (85). Furthermore, although butyrate and propionate can inhibit HDACs and promote the proliferation and differentiation of T_reg_ cells, acetate appears to lack this inhibitory activity towards HDACs (71).

Immune cells and cytokines are crucial in the development and regression of inflammatory responses. Inflammation is characterized by excessive infiltration of immune cells (e.g. macrophages, neutrophils), which subsequently release pro-inflammatory cytokines. Simultaneously, regression of inflammatory responses requires the release of anti-inflammatory factors (e.g. IL-10) by immune cells. Inulin and its metabolites can selectively support the development of Th1 and Th17 effector cells and IL-10^+^ T_reg_ cells, depending on the cytokine milieu and immunological context. SCFAs promote the differentiation of IL-10^+^ CD4^+^ T cells under a physiological state. Once the immune response is initiated, SCFAs turns to support the proliferation of T_eff_ cells, such as Th1 and Th17 cells (85). Therefore, inulin and its metabolites modulate the balance of the immune response, setting a reasonable “immune tension” that allows T cells to clear harmful substances but avoids exaggerating the level of tissue damage.

### Innate lymphoid cells

ILCs are an important subpopulation of natural immune cells. ILCs (like B cells and T cells) develop from common lymphoid progenitor cells, and share some common characteristics with T cells. However, ILCs do not express antigen-specific receptors (e.g., T-cell receptors, B-cell receptors). In addition, ILCs do not undergo thymic selection, clonal selection, or clonal expansion. Therefore, ILCs respond rapidly to tissue infection and pathogens, but the effector molecules produced are the same as those in Th cells. ILCs can be classified into four categories according to the cytokines they secrete: ILC1s secrete interferon (IFN)-γ; ILC2s secrete IL-5, IL-9, and IL-13; ILC3s secrete IL-22, IL-17A/F, and granulocyte macrophage-colony stimulating factor; regulatory ILC cells secrete IL-10 (86, 87). Multiple GPCR receptors are expressed on the surface of ILCs, and SCFAs have been found to activate GPCR receptors on ILCs and promote tissue repair and host defense, which contributes to regulating adaptive immunity (88–91).

Allergens lead to type-2 inflammatory responses, which are mediated by Th2 cells, ILC2s, and their secreted cytokines. Type-2 inflammatory responses can stimulate B cell proliferation to produce antibodies, mediate humoral immunity, participate in barrier immunity at mucosal surfaces, and play a part in counteracting parasitic infections and allergic diseases (92). An inulin fiber diet promotes type-2 immune responses after spirochete infection in an eosinophil-dependent manner (93). Furthermore, an increased activity of ILC2s plays a key part in asthma development (94), and inulin intervention has been reported to reduce the number of airway eosinophils and improve asthma by suppressing HDAC9 expression in people suffering from asthma (95). Furthermore, direct supplementation with SCFAs can also suppressed ILC2s and lung-related allergic reactions (96).

However, in contradiction to previous evidence, Arifuzzaman and colleagues showed that inulin increased systemic levels of bile acids (particularly cholic acid), which led to an increased IL-33 level via activation of the farnesoid X receptor (FXR) pathway. IL-33 secretion caused subsequent activation of ILC2s and the production of IL-5, leading to increased eosinophilia which exacerbated airway allergic responses (93). SCFAs-mediated FFAR2 expression has been shown to trigger phosphoinositide 3-kinase (PI3K), signal transducer and activator of transcription (Stat)3, Stat5, and mammalian target of rapamycin pathways to promote ILC2s proliferation. However, SCFAs also seem to inhibit the proliferation of ILC2s through a non-FFAR2-mediated mechanism (88). Thus, inulin may maintain optimal amounts of ILC2s in peripheral tissues to modulate type-2 immune responses during infections through multiple pathways. The immunological outcomes of consuming an inulin fiber diet are dependent upon the interactions between various microbiota-derived metabolites and different immunomodulatory pathways (93).

As a member of the IL-10 family, IL-22 has an important role in intestinal immune regulation. IL-22 has been reported to promote epithelial cell proliferation and induce the production of Reg3γ and other antimicrobial peptides (97). ILC3s, γδT lymphocytes and CD4^+^ T cells are the main cell types that secrete IL-22 in the gut (98). Inulin has been reported to promote colon epithelial remodeling by increasing γδT lymphocyte-induced IL-22 production (99). In addition, HFD consumption disrupts enterocyte proliferation, leading to an impaired intestinal barrier, low-grade inflammation, and metabolic syndrome. Conversely, inulin supplementation in a HFD impacts microbiota and promotes IL-22 expression in an ILC3s-dependent manner, which fortifies the intestine, thereby resulting in reduced microbiota encroachment and expression of pro-inflammatory genes (100). Several studies have also shown that SCFAs, which are products of the fermentation of DF (including inulin), promote the proliferation of ILC3s and CD4^+^ T cells and subsequent production of IL-22 through several mechanisms (101, 102).

### Monocytes and macrophages

Toll-like receptors (TLRs) recognize different pathogen-associated molecular patterns and trigger the production of pro-inflammatory factors in macrophages (103). However, sustained production of pro-inflammatory cytokines and chemokines can lead to disruption of immune homeostasis. As a TLR4 ligand, inulin activates TLR4 and regulates expression of inflammatory factors in monocytes (104). Butyrate has been shown to reverse the abnormal expression of ZO-1 and reduce LPS translocation as well as inhibit macrophage activation, pro-inflammatory cytokine production, and neutrophil infiltration, thereby reducing liver injury in rats (105). Similarly, Qiao et al. found that butyrate inhibits the production of TNF-α and IL-6 and myeloperoxidase activity by blocking NF-κB activation in Kupffer cells (106). In ulcerative colitis (UC), butyrate also inhibits NF-κB activation in macrophages and reduces mucosal inflammation (107). In a *Staphylococcus aureus*-induced mastitis model, intake of high-dose inulin was shown to inhibit HDAC3 by promoting butyrate production in mice, thereby activating the macrophage-mediated antimicrobial defense program (23). Moreover, butyrate administration in influenza-infected mice remodeled bone-marrow hematopoiesis, promoted production of Ly6c^-^ monocytes, and enhanced alternative macrophage activation, thereby inhibiting CXCL1 production, neutrophil recruitment, and the immune response during infection (28). Recently, the protective effect of butyrate was also observed in a peripheral blood mononuclear cell (PBMC) model of gout, in which butyrate downregulated the production of the pro-inflammatory cytokines IL-1β, IL-6, and IL-8 by inhibiting HDACs (108). In conclusion, these evidences suggest a protective role of inulin and SCFAs in regulating monocyte-macrophage-mediated protection in inflammatory responses and immune processes.

## Regulation of inulin and its metabolites of energy metabolism

DF can slow down the absorption of glucose as well as impede the uptake of dietary lipids and cholesterol to enhance satiety and improve insulin resistance. Due to these properties of DF, inulin shows unique protective effects on the metabolism of glucose, lipids and amino acids, involving multiple mechanisms and partly related to the immune response (17, 19, 24).

First, inulin has been shown to increase the number of L cells (which are responsible for secreting glucagon-like peptide 1 (GLP-1)), suggesting a potential effect of inulin on glucose metabolism (100). Second, maternal mice supplemented with inulin during pregnancy and lactation improved glucose tolerance in their offspring exposed to a maternal HFD by modulating DNA methylation and gene expression of *Wnt5a* and *Pi3k* (109). Inulin supplementation may alleviate hepatic steatosis by increasing adipose triglyceride lipase activity on hepatic lipid droplets and inhibiting expression of cannabinoid receptor-1 and patatin-like phospholipase-3 in the liver (110, 111).

Beek et al. traced inulin-derived SCFAs using a stable isotope tracer. They found that inulin intake increased plasma concentrations of propionate, butyrate, and acetate. This phenomenon may explain how inulin improves metabolism in obese men because SCFAs regulate the balance between the synthesis, oxidation and catabolism of fatty acids (18). Similarly, Guo et al. found that inulin-induced remodeling of the intestinal microbiota resulted in increased production of SCFAs that promoted expression of angiopoietin-like protein 4, which contributed to the improved metabolism of glucose and lipids (42). Furthermore, Zhao et al. demonstrated that inulin-induced SCFAs interact with FFARs expressed on L cells, promoting the secretion of intestinal peptides (including GLP-1 and fasting peptide YY (PYY)), thereby improving glucose metabolism and insulin resistance (112, 113).

Tryptophan is one of the eight essential amino acids. It is the only amino acid that contains an indole (bicyclic compound) structure. Tryptophan can be obtained only from the diet. The usual tryptophan metabolic pathways are kynurenine, indole, and serotonin (114). Tryptophan metabolites and kynurenine inhibit activated T cells, B cells and natural killer (NK) cells selectively under physiological status, and promote immunomodulatory effects by activating aryl hydrocarbon receptors (115). Indole-3-acetate can activate ILC3s (116). Indole can upregulate the expression of TJ protein mucin and anti-inflammatory factor protein IL-10 in IECs, and downregulate expression of the pro-inflammatory factor IL-8 (117, 118). Dietary supplementation with inulin can increase levels of alistipes and indole-3-acrylic acid, which are involved in tryptophan metabolism and improve obesity (47). Tryptophan metabolism is one of the key metabolic pathways affected by changes in intestinal microbiota and is closely related to intestinal immune regulation (115, 119). A metabolomic analysis targeting tryptophan metabolism showed that inulin intervention upregulated fecal levels of indole-3-acetate and kynurenine in rats with NAFLD, while downregulating levels of kynurenine and 5-hydroxyindoleacetic acid (21). Thus, inulin can mitigate pro-inflammatory effects.

## Role of inulin in disease

**Figure 2:**
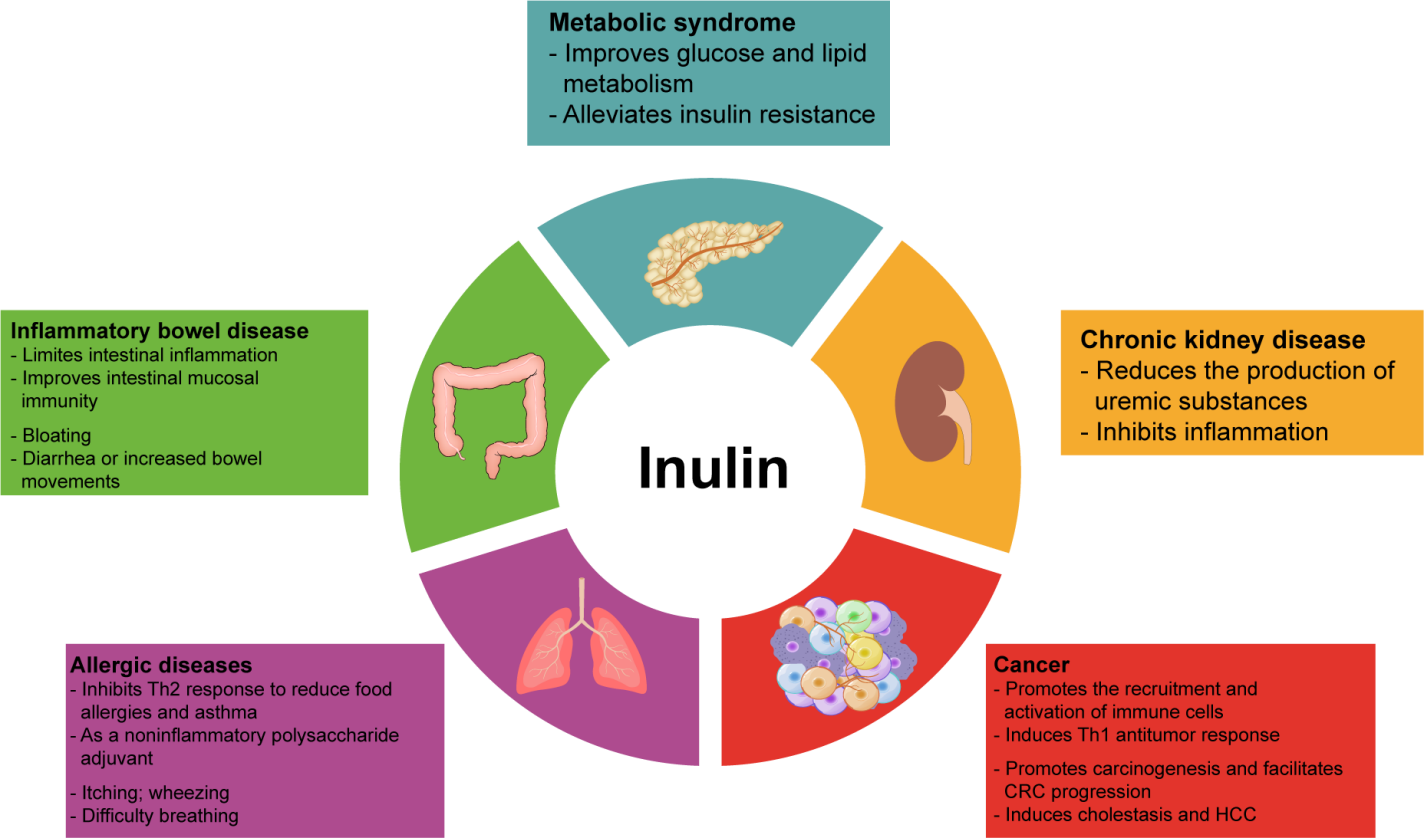
Overview of the involvement of inulin in disease. Inulin and its metabolites regulate energy metabolism and immune function, thus ameliorating various disease. However, inulin can also cause some side effects, such as nausea, bloating, flatulence, itching, and heartburn. In addition, people with inflammatory bowel disease or allergies should be more cautious about inulin intake to avoid serious adverse events.

Inulin can regulate the metabolism of glucose, lipids, and amino acids in addition to intestinal immune and systemic immunomodulatory effects. Its intestinal metabolites also exert beneficial functions. Therefore, inulin can improve the symptoms of many diseases, such as metabolic syndrome, IBD, and chronic kidney disease(CKD), associated with intestinal inflammation and intestinal dysbiosis, as well as allergic diseases and tumors related to immune imbalance (**Figure 2**). In **Table 3**, we have summarized some information about clinical trials on inulin use in different diseases, and the adverse effects caused by inulin.

**Table 3:** Effects of inulin ingestion in human studies.

Treatment	Duration	Subjects	Effects	Adverse effects	Reference
Inulin (10 g/d)	3 months	Diabetes *n*=27	Overall improvement in glycemic index, increased serum 10 g/d of butyric acid and propionic acid	–	(120)
Inulin (10 g/d)	6 weeks	Adults at risk of T2DM *n*=24	Increased *Bifidobacterium* abundance in gut	Mild gastrointestinal side effects, including bloating and loose stools	(121)
10 g/d of inulin or maltodextrin	8 weeks	Women suffering from obesity or depression *n*=45	No significant beneficial effects on depressive symptoms, gut permeability, or inflammatory biomarkers	Gastrointestinal complaints (flatulence, soft stools)	(122)
Inulin (60.2%) in 20-g formula	5 weeks	Healthy adults *n*=8	Suppressed postprandial glycemic response	–	(123)
Inulin-type fructans (16 g/d)	6 weeks	Patients with T2DM *n*=35	No significant effects on appetite hormones, subjective feeling of appetite or energy intake	Gastrointestinal symptoms (flatulence)	(124)
Inulin-type fructans (16 g/d)	6 weeks	Patients with T2DM *n*=25	A significant bifidogenic effect and increased fecal concentration of SCFAs	–	(19)
Butyrate (600 mg/d) + high-performance inulin (10 g/d)	45 days	Patients with T2DM *n*=60	A significant increase in expression of miR-146a and miR-9, and antioxidant capacity	–	(125)
45 g of milk powder with inulin and resistant dextrin	12 weeks	Older patients with T2DM *n*=99	Reduced systolic BP, diastolic BP, fasting and 2-h postprandial plasma glucose level, serum level of glycosylated proteins, and insulin resistance index; increased 2-h postprandial insulin level and β-cell function index	No serious adverse events	(126)
Inulin (1.7 g/d) in enriched seafood sticks (50 g/d)	12 weeks	Abdominally obese individuals *n*=120	Reduced postprandial atherogenic triglyceride concentrations and potential protection against T2DM	–	(127)
Inulin (16 g/d)	3 months	Obese participants *n*=61	BMI decrease, reduced liver stiffness and plasma levels of AST and cholesterol, and improved glucose intolerance	Rumbling, cramps, bloating and flatulence, which could be improved by physical activity	(128)
16 g/d inulin	3 months	Obese patients *n*=106	Improved bodyweight, AST level and insulinemia, decreased abundance of *Desulfovibrio* and *Clostridium*, and increased abundance of *Bifidobacterium*, but without gut microbiota changes or metabolic improvements after metformin treatment	Nausea, cramp, reflux and rumbling	(43)
Inulin-propionate ester (20 g/d)	42 days	Overweight and obese adults not suffering from diabetes mellitus *n*=12	Improved insulin resistance, increased abundance of Actinobacteria and decreased abundance of Clostridia	Stomach discomfort, nausea, bloating, flatulence, belching, heartburn	(45)
10 g/d of a mixture of inulin and oligofructose	12 weeks	Patients undergoing continuous ambulatory peritoneal dialysis *n*=16	Changes in the composition of intestinal microbiota, reduction of the serum levels of uric acid, and increase in fecal degradation of uric acid	–	(129)
Inulin-type fructans (10 g/d)	3 months	Patients undergoing continuous ambulatory peritoneal dialysis *n*=22	Altered composition of intestinal microbiota	No adverse effects	(130)

T2DM, type 2 diabetes mellitus; SCFAs, short-chain fatty acids; BP, blood pressure; BMI, body mass index; AST, aspartate transaminase.

### Metabolic syndrome

Metabolic syndrome is largely caused by physical inactivity and excess caloric intake. Patients with metabolic syndrome often have abdominal obesity, insulin resistance, hyperglycemia, hyperlipidemia and hypertension. Growing evidence suggests that obesity and metabolic disorders are associated with ecological dysbiosis of the gut microbiota, and that increased intake of DF is beneficial in improving ecological dysbiosis (131, 132). Thus, dietary inulin is a potential agent for improving disorders of glucose and lipid metabolism (20, 125, 128).

Studies have demonstrated that inulin intake modulates ecological dysbiosis, reduces the level of fasting glucose, attenuates insulin resistance, and improves lipid disorders (126, 127). However, some patients may suffer from mild gastrointestinal discomfort, including bloating and loose stools (45, 120, 121, 123). In a mouse model of a Western diet (42% of calories from fat, 43% of calories from carbohydrates, and 15% of calories from protein)-induced dysbiosis colonized with human vegan microbiota, inulin supplementation rendered a shift from protein hydrolysis to glycolytic fermentation of the gut microbiota. This action resulted in fewer sulfur-containing compounds and more SCFAs, which contributed to improved lipid denaturation and glucose homeostasis (133). The same improvement in the metabolism of glucose and lipids was also observed in *ob/ob* mice upon inulin treatment (42). Inulin also shows a significant improvement in type I diabetes mellitus (T1DM) in addition to T2DM. Disruption of the gut barrier leads to activation of pancreatic islet-reactive T cells and triggers autoimmune T1DM (134, 135), and diet is one of the most important factors in affecting gut homeostasis. Several studies have shown that an inulin-rich diet can promote a beneficial gut microbiota composition, and increase expression of TJ proteins and mucins, thereby preventing and/or treating T1DM (55, 136, 137). The improvement of T1DM by inulin is dependent on its modulation of the intestinal metabolic profile because the fermentation of inulin by gut microbiota promotes SCFAs production and a subsequent increase in the number of Foxp3^+^ T_reg_ and IL-10^+^ Tr1 cells, which may limit activation of pancreatic islet- reactive T cells (138).

Intriguingly, another study found that mice supplemented with inulin undertook more locomotive activity than those supplemented with cellulose. Those data suggested that inulin intake intensified the willingness of mice to exercise and promoted energy expenditure in obese mice (139). However, the mechanisms behind these changes are largely unknown and may be related to the regulation of the nervous system by inulin metabolites. Guo et al. found that inulin could modulate neurological disorders through the microbiome-gut-brain axis (140). In addition, Shulman and colleagues reported that a HFD induced an increase in acetate production in the intestine of mice, and then the increased acetate level led to activation of the parasympathetic nervous system and promoted secretion of growth hormone-releasing peptide and glucose-stimulated insulin. In that study, direct stimulation of isolated pancreatic islets with acetate failed to promote insulin secretion. However, these changes were not observed when the parasympathetic nerves were cut off, which indicated that parasympathetic nerves in the gut-brain-pancreatic-β-cell axis might be involved in the regulation of inulin or its metabolites (141). However, other researchers have reported no significant effect on appetite after inulin intake

### Inflammatory bowel disease

IBD includes Crohn’s disease and UC. Chronic intestinal inflammation is the typical feature of IBD. IBD development is associated with environmental factors, genetic conditions, faults in the immune system, and changes in the microbiota (142). Inulin has been reported to limit intestinal inflammation, modulate the intestinal microbiome, and improve intestinal barrier function. A randomized controlled trial supported the notion that oligofructose-enriched inulin can improve gastrointestinal symptoms in patients with active UC without significant side effects (143). Therefore, inulin is also being used increasingly for IBD treatment (144, 145).

The ameliorative effect of inulin on IBD is related mainly to its: reshaping of intestinal microbiota structure; promoting the growth of beneficial bacteria; inhibiting expression of inflammatory factors; improving the intestinal mucosal barrier. As mentioned above, inulin significantly increased the abundance of beneficial bacteria such as *Bifidobacterium rhamnosus*. In an animal model induced by DSS, inulin combined with *Lactobacillus rhamnosus* increased the abundance and diversity of intestinal microbiota, decreased expression of pro-inflammatory cytokines, and relieved UC (146). In a study comparing the differences between inulin and another type of DF, the authors found that inulin had a modulatory effect on the microbiota of mice with DSS-induced colitis, reduced expression of pro-inflammatory cytokines significantly, and improved intestinal barrier function (147). Those results support that the notion that DFs (especially inulin) are promising dietary supplements to alleviate intestinal inflammation. In addition, inulin can be used as an immune- system modulator for the treatment and management of IBD, and its mechanism is related to the promotion of secretion of antimicrobial peptides and improvement of intestinal mucosal immunity (148).

Results in animal IBD models and humans suggest that inulin intake can help to improve the intestinal mucosal barrier and suppress intestinal inflammation (149), but some research teams have reached opposite conclusions. For example, Armstrong and colleagues found that unfermented inulin induced secretion of pro-inflammatory cytokines in a subset of IBD intestinal biopsies cultured *ex vivo* (150). In several other animal studies, researchers have found that dietary supplementation with inulin may be beneficial for low-grade inflammation and associated metabolic disease, but that it also exacerbates the severity of DSS-induced acute colitis (151–153). Furthermore, treatment with an “antibiotic cocktail” led to intestinal ecological dysregulation and induced colitis in mice, whereas supplementation with inulin-type fructans delayed the recovery of this antibiotic-induced intestinal inflammation and decreased the recovery of T_reg_ and B cells in the lamina propria. Moreover, although supplementation with inulin-type fructans inhibited expression of certain pro-inflammatory genes in the colon (e.g., inducible nitric oxide synthase, TNF-α), it also reduced sIgA secretion in the colon. Inulin also increased the serum level of LPS, reduced secretion of the anti-inflammatory mediator transforming growth factor-β1, and promoted secretion of the pro-inflammatory cytokine IL-17A (154). In a study on the anti-tumor effect of inulin, inulin promoted the infiltration of γδ T cells and production of IFN-γ in tumors, but also led to expression of several inflammation-related genes in IECs, including TNF-α, cyclooxygenase-9, and MMP-9, thereby exacerbating inflammation in the intestine, but this seems to be associated with immune surveillance. Inulin also triggered the expression of macrophage inflammatory protein-2, IL-22 and the transcription factor Foxp3 in CD45^+^ cells in the lamina propria, and these were beneficial in suppressing inflammation. Those results suggest that an inulin diet triggers activation of γδ T cells in epithelial lymphocytes and immune surveillance in IECs, as well as induction of tissue repair signals and tolerance in cells of the lamina propria (69).

Overall, inulin is beneficial for IBD because it reshapes the intestinal microbiota structure, suppresses intestinal inflammation, and improves intestinal cellular and mucosal immunity. However, the gas produced by fermentation of inulin may aggravate the gastrointestinal symptoms of patients, thus limiting its beneficial effects (155). In two randomized controlled clinical trials, inulin ingestion did not change appetite, intestinal permeability, or levels of inflammatory biomarkers, but caused flatulence and soft stools (122, 124).

### Chronic kidney disease

Urea accumulation associated with CKD can affect the composition of the gut microbiome and increase the permeability of the intestinal epithelial barrier. If the intestinal barrier is breached, uremic substances, including indole sulfate, para-cresol sulfate, and trimethylamine N-oxide (TMAO), can lead to endotoxemia and systemic inflammation (156, 157). Recently, modification of the gut flora by supplementation with prebiotics has been considered to be a potential therapeutic strategy to reduce uremic toxins of intestinal origin and inflammation. For example, inulin supplementation changed the composition of intestinal microbiota, reduced serum levels of uric acid, and increased degradation of fecal uric acid in patients with renal failure (129). Moreover, intake of inulin-type fructans limited the production of indoles (precursors of indoxyl sulfate) in patients undergoing peritoneal dialysis (158). Similar results were observed by Mitrović et al. They found that inulin treatment reduced the serum level of indoxyl sulfate, improved the glomerular filtration rate, and reduced the level of high sensitivity C-reactive protein levels by altering the gut microbiota composition in patients with CKD (159). In addition, long-term consumption of inulin-containing fructan water reduced serum levels of glucose, total cholesterol, uric acid and creatine kinase in mouse offspring, suggesting that inulin-type fructans contribute to a reduced risk of kidney disease (160).

However, in another study, intervention with inulin-type fructans (10 g/day) for 3 months altered composition of gut microbiome, but did not reduce the plasma TMAO level in patients undergoing peritoneal dialysis (130). This observation may be related to the duration and dose of the intervention. Furthermore, according to the results of a prospective cohort study, higher dietary inulin intake also failed to reduce the incidence of CKD and cardiovascular disease in the population, but prevented hypertension and T2DM, which are major risk factors for cardiovascular and renal events (161). Therefore, given that inulin showed an overall benefit or a neutral effect, inulin is considered to be a safe and reliable strategy to improve the uremic toxin and micro-inflammatory state in patients with CKD (159).

### Allergic diseases

ILC2s and Th2 cells are among the key effector cells in allergic diseases, and the cytokines they secrete (IL-4, IL-5, IL-13) mediate the allergic immune response (162). Inulin and its intestinal metabolites (SCFAs) may be involved in mediating the amelioration of allergic diseases by regulating ILC2s and Th2 cells.

Several studies have shown that inulin supplementation in mice during gestation or lactation induced the growth of beneficial bacteria in the intestine of maternal mice. These beneficial bacteria could also be transferred to their offspring, enhance their intestinal barrier function, and increase the number of B-cell and T_reg_ subpopulations in lymph nodes. These actions shaped a more tolerogenic immune environment that suppressed Th2 responses to alleviate food allergy (163, 164). Furthermore, in airway allergic responses, propionate ameliorates inflammation by altering bone-marrow hematopoiesis in mice via FFAR3, promotes the production of DC precursors, and inhibits the differentiation capacity of Th2 cells (72). Several studies have demonstrated that inulin diets exhibit benefits for allergic diseases (including asthma), but inulin itself can cause rare allergic reactions (e.g., itching, rash, swelling, wheezing, difficulty in breathing, unconsciousness) (165–167).

In addition, inulin (especially delta inulin) has been used as an adjuvant to enhance the immune response (168). Venom immunotherapy is effective in improving anaphylactic reactions to stings from Hymenoptera spp, but it can also cause severe (and even life-threatening) immune reactions. Plant-based polysaccharide delta inulin is a new adjuvant with low reactogenicity that can enhance vaccine immunogenicity and antigen-sparing. A randomized controlled trial reported the benefit of delta inulin as an immune adjuvant in patients with bee-venom allergy, and found that delta inulin increased the levels of specific IgG_4_ significantly during the early induction phase (169). In conclusion, even though inulin may cause allergic reactions, the function of inulin as a dietary supplement to alleviate allergic diseases (e.g., food allergies, asthma) or as an adjuvant to enhance vaccine efficacy has been demonstrated widely and utilized.

### Cancer

Dietary supplementation with whole grains and DF usually reduces the incidence of tumors as well as the risk of postoperative oncologic complications and tumor-related mortality (170, 171). Studies have observed the tumor growth inhibitory effects of inulin, though the mechanisms remain largely unexplored (172, 173). Nevertheless, several mechanisms pertaining to the activity and regulation of inulin in anti-tumor immunity have been elucidated in recent years.

Perhaps the anti-tumor effects of inulin rely largely on its ability to promote immune cell recruitment to the tumor microenvironment. Two studies found increased infiltration of immune cells in the tumor bed after supplementation with an inulin-rich diet (69, 70). Upon subcutaneous injection of a syngeneic B16- ovalbumin melanoma tumor, inulin uptake promoted infiltration of CD4^+^ and CD8^+^ T cells and increased IFN-γ production, thereby triggering an effective Th1 anti-tumor response and inhibiting tumor growth. Meanwhile, inulin treatment increased expression of chemokines (CCL4, CCL8), inflammatory vesicle-related genes (TLR3, TLR7) and antigen presentation-related genes (CD40, Stat1, ICOS), induced anti-tumor immunity, and inhibited the growth of colon tumors. Moreover, either alone or in combination with SCFAs, inulin affected tumor growth, indicating that the anti-tumor effects of inulin were not dependent on SCFAs (70). Notably, in addition to B16-OVA melanoma tumors, the anti-tumor effects of inulin were also confirmed in tumor models of MCA205 fibrosarcoma and MC38 colorectal cancer (CRC) cell lines, and such effects were associated with the response of Th1 cells (69). In addition, γδ T cells are unconventional T cells that recognize metabolism-related molecules and have potent anti-tumor activity. Inulin can activate γδ T cells via γδ T cell receptor signaling, and promote IFN-γ production (174). In mice with 1,2-dimethylhydrazine-induced colon cancer, the amelioration of colon cancer in mice by inulin involved modulation of Janus kinase-1/β-catenin signaling (175).

In liver-associated tumors, the anti-tumor effect of inulin is associated with its metabolites and subsequent immunomodulation. In mice with hepatocellular carcinoma (HCC), an increased acetate level by fecal-bacterial transplantation or direct administration of acetate inhibited the activity of HDACs, increased acetylation of sex-determining region Y-box transcription factor 13 (Sox13) at site K30, and decreased expression of Sox13, thereby reducing IL-17A production by ILC3s and retarding tumor growth. In addition, a combination of acetate with blockade of programmed death (PD)-1/PD-1 ligand promoted anti-tumor immunity significantly and enhanced the treatment efficacy of PD-1 (176).

Inulin has shown protective and tumor-suppressive effects in most CRC studies, but other reports have indicated that inulin intake promotes CRC development. The reason for this discrepancy may be due to differences in gut microbial composition. Inulin supplementation led to increased colonization of polyketide synthase-positive (*pks^+^*) *E. coli* strain NC101, whereas *pks^+^ Escherichia coli* can promote carcinogenesis and facilitate CRC progression through the production of colistin (a genotoxin that induces double-stranded DNA breaks) (177). Therefore, given the prevalence of *pks^+^ E. coli* in healthy and CRC populations, individuals colonized with *pks^+^* bacteria should use inulin with caution (178). Furthermore, supplementation of inulin can induce cholestasis and HCC, which may be due to inulin fermentation (179).

## Conclusions

DFs are indispensable supplements in daily life. Inulin and its metabolites (SCFAs) have key roles in lowering blood glucose, reducing bodyweight, and improving insulin resistance. The fermentation of inulin by intestinal microbiota can promote the proliferation of beneficial flora, regulate intestinal pH and maintain the homeostasis of the intestinal ecological environment. Therefore, dietary intake of inulin may serve as a simple but effective way to improve intestinal and systemic immune function and prevent diseases, and sufficient intake of inulin fiber is recommended. However, inulin ingestion may cause gastrointestinal symptoms, allergies or even more serious adverse effects, so it should be consumed under the supervision of healthcare professionals.

## Author contributions

LZ conceptualized the manuscript, WS collected the literature and drafted the manuscript, LZ and GJ revised the manuscript. All authors contributed to the article and approved the submitted version.

## References

[1] CAC. Codex Alimentarius Commission Guidelines on Nutrition Labelling CAC/GL 2—1985 as Last Amended 2021. (2021). Rome: FAO. ICACJFWFS.

[2] Gasaly N de Vos P Hermoso MA. Impact of bacterial metabolites on gut barrier function and host immunity: A focus on bacterial metabolism and its relevance for intestinal inflammation. Front Immunol (2021) 12:658354. doi: 10.3389/fimmu.2021.658354 34122415 PMC8187770

[3] Larsson E Tremaroli V Lee YS Koren O Nookaew I Fricker A. Analysis of gut microbial regulation of host gene expression along the length of the gut and regulation of gut microbial ecology through myd88. Gut (2012) 61(8):1124–31. doi: 10.1136/gutjnl-2011-301104 PMC3388726 22115825

[4] Kelly G. Inulin-type prebiotics–a review: part 1. Altern Med Rev (2008) 13(4):315–29. 19152479

[5] Niness KR. Inulin and oligofructose: what are they? J Nutr (1999) 129(7 Suppl):1402S–6S. doi: 10.1093/jn/129.7.1402S 10395607

[6] Shoaib M Shehzad A Omar M Rakha A Raza H Sharif HR. Inulin: properties, health benefits and food applications. Carbohydr Polym (2016) 147:444–54. doi: 10.1016/j.carbpol.2016.04.020 27178951

[7] U.S. Department of Health and Human Services. United States Department of Agriculture. Dietary guidelines for Americans, 2020-2025. (U.S. Department of Agriculture). (2020). Available at: https://Www.Dietaryguidelines.Gov.

[8] Food and Drug Administration. Science review of isolated and synthetic non-digestible carbohydrates. (U.S. Food and Drug Administration). (2016) Available at: https://Www.Fda.Gov/Media/101853/Download.

[9] Yuan C Wang S Gebeyew K Yang X Tang S Zhou C. A low-carbon high inulin diet improves intestinal mucosal barrier function and immunity against infectious diseases in goats. Front Vet Sci (2022) 9:1098651. doi: 10.3389/fvets.2022.1098651 36713857 PMC9874328

[10] Roberfroid M Slavin J. Nondigestible oligosaccharides. Crit Rev Food Sci Nutr (2000) 40(6):461–80. doi: 10.1080/10408690091189239 11186236

[11] Bornet FR. Undigestible sugars in food products. Am J Clin Nutr (1994) 59(3 Suppl):763S–9S. doi: 10.1093/ajcn/59.3.763S 8116563

[12] McBain AJ Macfarlane GT. Investigations of bifidobacterial ecology and oligosaccharide metabolism in a three-stage compound continuous culture system. Scand J Gastroenterol Suppl (1997) 222:32–40. doi: 10.1080/00365521.1997.11720715 9145444

[13] Gill SR Pop M Deboy RT Eckburg PB Turnbaugh PJ Samuel BS. Metagenomic analysis of the human distal gut microbiome. Science (2006) 312(5778):1355–9. doi: 10.1126/science.1124234 PMC3027896 16741115

[14] Yu X Gurry T Nguyen LTT Richardson HS Alm EJ. Prebiotics and community composition influence gas production of the human gut microbiota. mBio (2020) 11(5). doi: 10.1128/mBio.00217-20 PMC7482059 32900799

[15] Rahat-Rozenbloom S Fernandes J Cheng J Wolever TMS. Acute increases in serum colonic short-chain fatty acids elicited by inulin do not increase glp-1 or pyy responses but may reduce ghrelin in lean and overweight humans. Eur J Clin Nutr (2017) 71(8):953–8. doi: 10.1038/ejcn.2016.249 PMC5423780 27966574

[16] Nakayama Y Kawasaki N Tamiya T Anzai S Toyohara K Nishiyama A. Comparison of the prebiotic properties of native chicory and synthetic inulins using swine fecal cultures. Biosci Biotechnol Biochem (2020) 84(7):1486–96. doi: 10.1080/09168451.2020.1749553 32281519

[17] Tarini J Wolever TM. The fermentable fibre inulin increases postprandial serum short-chain fatty acids and reduces free-fatty acids and ghrelin in healthy subjects. Appl Physiol Nutr Metab (2010) 35(1):9–16. doi: 10.1139/H09-119 20130660

[18] van der Beek CM Canfora EE Kip AM Gorissen SHM Olde Damink SWM van Eijk HM. The prebiotic inulin improves substrate metabolism and promotes short-chain fatty acid production in overweight to obese men. Metabolism (2018) 87:25–35. doi: 10.1016/j.metabol.2018.06.009 29953876

[19] Birkeland E Gharagozlian S Birkeland KI Valeur J Mage I Rud I. Correction to: prebiotic effect of inulin−Type fructans on faecal microbiota and short−Chain fatty acids in type 2 diabetes: A randomised controlled trial. Eur J Nutr (2020) 59(7):3339–40. doi: 10.1007/s00394-020-02314-0 PMC7501097 32440730

[20] Birkeland E Gharagozlian S Birkeland KI Valeur J Mage I Rud I. Prebiotic effect of inulin-type fructans on faecal microbiota and short-chain fatty acids in type 2 diabetes: A randomised controlled trial. Eur J Nutr (2020) 59(7):3325–38. doi: 10.1007/s00394-020-02282-5 PMC7501097 32440730

[21] Yang Z Su H Lv Y Tao H Jiang Y Ni Z. Inulin intervention attenuates hepatic steatosis in rats via modulating gut microbiota and maintaining intestinal barrier function. Food Res Int (2023) 163:112309. doi: 10.1016/j.foodres.2022.112309 36596207

[22] He J Xie H Chen D Yu B Huang Z Mao X. Synergetic responses of intestinal microbiota and epithelium to dietary inulin supplementation in pigs. Eur J Nutr (2021) 60(2):715–27. doi: 10.1007/s00394-020-02284-3 32435994

[23] Zhao C Bao L Zhao Y Wu K Qiu M Feng L. A fiber-enriched diet alleviates staphylococcus aureus-induced mastitis by activating the hdac3-mediated antimicrobial program in macrophages via butyrate production in mice. PloS Pathog (2023) 19(1):e1011108. doi: 10.1371/journal.ppat.1011108 36656870 PMC9888710

[24] Nakajima H Nakanishi N Miyoshi T Okamura T Hashimoto Y Senmaru T. Inulin reduces visceral adipose tissue mass and improves glucose tolerance through altering gut metabolites. Nutr Metab (Lond) (2022) 19(1):50. doi: 10.1186/s12986-022-00685-1 35902903 PMC9331483

[25] Louis P Duncan S Sheridan P Walker A Flint H. Microbial lactate utilisation and the stability of the gut microbiome. Gut Microbiome (2022) 3. doi: 10.1017/gmb.2022.3 PMC11406415 39295779

[26] Tao S Bai Y Zhou X Zhao J Yang H Zhang S. *In vitro* fermentation characteristics for different ratios of soluble to insoluble dietary fiber by fresh fecal microbiota from growing pigs. ACS Omega (2019) 4(12):15158–67. doi: 10.1021/acsomega.9b01849 PMC6751720 31552361

[27] Muthyala SDV Shankar S Klemashevich C Blazier JC Hillhouse A Wu CS. Differential effects of the soluble fiber inulin in reducing adiposity and altering gut microbiome in aging mice. J Nutr Biochem (2022) 105:108999. doi: 10.1016/j.jnutbio.2022.108999 35346831

[28] Trompette A Gollwitzer ES Pattaroni C Lopez-Mejia IC Riva E Pernot J. Dietary fiber confers protection against flu by shaping ly6c(-) patrolling monocyte hematopoiesis and cd8(+) T cell metabolism. Immunity (2018) 48(5):992–1005 e8. doi: 10.1016/j.immuni.2018.04.022 29768180

[29] Zeng H Umar S Rust B Lazarova D Bordonaro M. Secondary bile acids and short chain fatty acids in the colon: A focus on colonic microbiome, cell proliferation, inflammation, and cancer. Int J Mol Sci (2019) 20(5). doi: 10.3390/ijms20051214 PMC6429521 30862015

[30] Donohoe DR Garge N Zhang X Sun W O’Connell TM Bunger MK. The microbiome and butyrate regulate energy metabolism and autophagy in the mamMalian colon. Cell Metab (2011) 13(5):517–26. doi: 10.1016/j.cmet.2011.02.018 PMC3099420 21531334

[31] Cummings JH Pomare EW Branch WJ Naylor CP Macfarlane GT. Short chain fatty acids in human large intestine, portal, hepatic and venous blood. Gut (1987) 28(10):1221–7. doi: 10.1136/gut.28.10.1221 PMC1433442 3678950

[32] Koh A De Vadder F Kovatcheva-Datchary P Backhed F. From dietary fiber to host physiology: short-chain fatty acids as key bacterial metabolites. Cell (2016) 165(6):1332–45. doi: 10.1016/j.cell.2016.05.041 27259147

[33] Ikeda T Nishida A Yamano M Kimura I. Short-chain fatty acid receptors and gut microbiota as therapeutic targets in metabolic, immune, and neurological diseases. Pharmacol Ther (2022) 239:108273. doi: 10.1016/j.pharmthera.2022.108273 36057320

[34] Round JL Mazmanian SK. Inducible foxp3+ Regulatory T-cell development by a commensal bacterium of the intestinal microbiota. Proc Natl Acad Sci U.S.A. (2010) 107(27):12204–9. doi: 10.1073/pnas.0909122107 PMC2901479 20566854

[35] Nilsson NE Kotarsky K Owman C Olde B. Identification of a free fatty acid receptor, ffa2r, expressed on leukocytes and activated by short-chain fatty acids. Biochem Biophys Res Commun (2003) 303(4):1047–52. doi: 10.1016/s0006-291x(03)00488-1 12684041

[36] Becattini S Taur Y Pamer EG. Antibiotic-induced changes in the intestinal microbiota and disease. Trends Mol Med (2016) 22(6):458–78. doi: 10.1016/j.molmed.2016.04.003 PMC4885777 27178527

[37] Turnbaugh PJ Ley RE Mahowald MA Magrini V Mardis ER Gordon JI. An obesity-associated gut microbiome with increased capacity for energy harvest. Nature (2006) 444(7122):1027–31. doi: 10.1038/nature05414 17183312

[38] Ley RE Turnbaugh PJ Klein S Gordon JI. Microbial ecology: human gut microbes associated with obesity. Nature (2006) 444(7122):1022–3. doi: 10.1038/4441022a 17183309

[39] Le Bastard Q Chapelet G Javaudin F Lepelletier D Batard E Montassier E. The effects of inulin on gut microbial composition: A systematic review of evidence from human studies. Eur J Clin Microbiol Infect Dis (2020) 39(3):403–13. doi: 10.1007/s10096-019-03721-w 31707507

[40] Zhang Q Yu H Xiao X Hu L Xin F Yu X. Inulin-type fructan improves diabetic phenotype and gut microbiota profiles in rats. PeerJ (2018) 6:e4446. doi: 10.7717/peerj.4446 29507837 PMC5835350

[41] Guo Y Yu Y Li H Ding X Li X Jing X. Inulin supplementation ameliorates hyPeruricemia and modulates gut microbiota in uox-knockout mice. Eur J Nutr (2021) 60(4):2217–30. doi: 10.1007/s00394-020-02414-x PMC8137640 33104864

[42] Guo J Zhang M Wang H Li N Lu Z Li L. Gut microbiota and short chain fatty acids partially mediate the beneficial effects of inulin on metabolic disorders in obese ob/ob mice. J Food Biochem (2022) 46(5):e14063. doi: 10.1111/jfbc.14063 35128673

[43] Hiel S GianFrancesco MA Rodriguez J Portheault D Leyrolle Q Bindels LB. Link between gut microbiota and health outcomes in inulin -treated obese patients: lessons from the food4gut multicenter randomized placebo-controlled trial. Clin Nutr (2020) 39(12):3618–28. doi: 10.1016/j.clnu.2020.04.005 32340903

[44] Yang X He F Zhang Y Xue J Li K Zhang X. Inulin ameliorates alcoholic liver disease via suppressing lps-tlr4-mpsi axis and modulating gut microbiota in mice. Alcohol Clin Exp Res (2019) 43(3):411–24. doi: 10.1111/acer.13950 30589437

[45] Chambers ES Byrne CS Morrison DJ Murphy KG Preston T Tedford C. Dietary supplementation with inulin-propionate ester or inulin improves insulin sensitivity in adults with overweight and obesity with distinct effects on the gut microbiota, plasma metabolome and systemic inflammatory responses: A randomised cross-over trial. Gut (2019) 68(8):1430–8. doi: 10.1136/gutjnl-2019-318424 PMC6691855 30971437

[46] Park JH Song WS Lee J Jo SH Lee JS Jeon HJ. An integrative multiomics approach to characterize prebiotic inulin effects on faecalibacterium prausnitzii. Front Bioeng Biotechnol (2022) 10:825399. doi: 10.3389/fbioe.2022.825399 35252133 PMC8894670

[47] Wu Z Du Z Tian Y Liu M Zhu K Zhao Y. Inulin accelerates weight loss in obese mice by regulating gut microbiota and serum metabolites. Front Nutr (2022) 9:980382. doi: 10.3389/fnut.2022.980382 36245535 PMC9554005

[48] Odenwald MA Turner JR. The intestinal epithelial barrier: A therapeutic target? Nat Rev Gastroenterol Hepatol (2017) 14(1):9–21. doi: 10.1038/nrgastro.2016.169 27848962 PMC5554468

[49] Beumer J Clevers H. Cell fate specification and differentiation in the adult mamMalian intestine. Nat Rev Mol Cell Biol (2021) 22(1):39–53. doi: 10.1038/s41580-020-0278-0 32958874

[50] Lueschow SR McElroy SJ. The paneth cell: the curator and defender of the immature small intestine. Front Immunol (2020) 11:587. doi: 10.3389/fimmu.2020.00587 32308658 PMC7145889

[51] Pang DJ Huang C Chen ML Chen YL Fu YP Paulsen BS. Characterization of inulin-type fructan from platycodon grandiflorus and study on its prebiotic and immunomodulating activity. Molecules (2019) 24(7). doi: 10.3390/molecules24071199 PMC6479354 30934739

[52] Zou YF Zhang YY Zhu ZK Fu YP Paulsen BS Huang C. Characterization of inulin-type fructans from two species of radix codonopsis and their oxidative defense activation and prebiotic activities. J Sci Food Agric (2021) 101(6):2491–9. doi: 10.1002/jsfa.10875 33063324

[53] Ze X Duncan SH Louis P Flint HJ. Ruminococcus bromii is a keystone species for the degradation of resistant starch in the human colon. ISME J (2012) 6(8):1535–43. doi: 10.1038/ismej.2012.4 PMC3400402 22343308

[54] Russell WR Gratz SW Duncan SH Holtrop G Ince J Scobbie L. High-protein, reduced-carbohydrate weight-loss diets promote metabolite profiles likely to be detrimental to colonic health. Am J Clin Nutr (2011) 93(5):1062–72. doi: 10.3945/ajcn.110.002188 21389180

[55] Chen K Chen H Faas MM de Haan BJ Li J Xiao P. Specific inulin-type fructan fibers protect against autoimmune diabetes by modulating gut immunity, barrier function, and microbiota homeostasis. Mol Nutr Food Res (2017) 61(8). doi: 10.1002/mnfr.201601006 28218451

[56] Shan M Gentile M Yeiser JR Walland AC Bornstein VU Chen K. Mucus enhances gut homeostasis and oral tolerance by delivering immunoregulatory signals. Science (2013) 342(6157):447–53. doi: 10.1126/science.1237910 PMC4005805 24072822

[57] Zou YF Li CY Fu YP Feng X Peng X Feng B. Restorative effects of inulin from codonopsis pilosula on intestinal mucosal immunity, anti-inflammatory activity and gut microbiota of immunosuppressed mice. Front Pharmacol (2022) 13:786141. doi: 10.3389/fphar.2022.786141 35237158 PMC8882912

[58] Beisner J Filipe Rosa L Kaden-Volynets V Stolzer I Gunther C Bischoff SC. Prebiotic inulin and sodium butyrate attenuate obesity-induced intestinal barrier dysfunction by induction of antimicrobial peptides. Front Immunol (2021) 12:678360. doi: 10.3389/fimmu.2021.678360 34177920 PMC8226265

[59] Peng L Li ZR Green RS Holzman IR Lin J. Butyrate enhances the intestinal barrier by facilitating tight junction assembly via activation of amp-activated protein kinase in caco-2 cell monolayers. J Nutr (2009) 139(9):1619–25. doi: 10.3945/jn.109.104638 PMC2728689 19625695

[60] Xie Q Mu K Chen C Gu S Luo D Fu W. The high dose of inulin exacerbated food allergy through the excess accumulation of short-chain fatty acids in a babl/C mouse model. Int J Biol Macromol (2023) 230:123234. doi: 10.1016/j.ijbiomac.2023.123234 36642358

[61] He Y Peng X Liu Y Wu Q Zhou Q Huang Y. Long-term maternal intake of inulin exacerbated the intestinal damage and inflammation of offspring rats in a dss-induced colitis model. Food Funct (2022) 13(7):4047–60. doi: 10.1039/d1fo03675k 35315466

[62] Van Kaer L Olivares-Villagomez D. Development, homeostasis, and functions of intestinal intraepithelial lymphocytes. J Immunol (2018) 200(7):2235–44. doi: 10.4049/jimmunol.1701704 PMC5863587 29555677

[63] Matsunaga Y Clark T Wanek AG Bitoun JP Gong Q Good M. Intestinal il-17r signaling controls secretory iga and oxidase balance in citrobacter rodentium infection. J Immunol (2021) 206(4):766–75. doi: 10.4049/jimmunol.2000591 PMC8663204 33431657

[64] Kim CH Park J Kim M. Gut microbiota-derived short-chain fatty acids, T cells, and inflammation. Immune Netw (2014) 14(6):277–88. doi: 10.4110/in.2014.14.6.277 PMC4275385 25550694

[65] Schiweck C Edwin Thanarajah S Aichholzer M Matura S Reif A Vrieze E. Regulation of cd4(+) and cd8(+) T cell biology by short-chain fatty acids and its relevance for autoimmune pathology. Int J Mol Sci (2022) 23(15). doi: 10.3390/ijms23158272 PMC9368239 35955407

[66] Smith PM Howitt MR Panikov N Michaud M Gallini CA Bohlooly YM. The microbial metabolites, short-chain fatty acids, regulate colonic treg cell homeostasis. Science (2013) 341(6145):569–73. doi: 10.1126/science.1241165 PMC3807819 23828891

[67] Takahashi D Hoshina N Kabumoto Y Maeda Y Suzuki A Tanabe H. Microbiota-derived butyrate limits the autoimmune response by promoting the differentiation of follicular regulatory T cells. EBioMedicine (2020) 58:102913. doi: 10.1016/j.ebiom.2020.102913 32711255 PMC7387783

[68] Kassayova M Bobrov N Strojny L Orendas P Demeckova V Jendzelovsky R. Anticancer and immunomodulatory effects of lactobacillus plantarum ls/07, inulin and melatonin in nmu-induced rat model of breast cancer. Anticancer Res (2016) 36(6):2719–28. 27272781

[69] Boucher E Plazy C Richard ML Suau A Mangin I Cornet M. Inulin prebiotic reinforces host cancer immunosurveillance via ɣdelta T cell activation. Front Immunol (2023) 14:1104224. doi: 10.3389/fimmu.2023.1104224 36875124 PMC9981629

[70] Li Y Elmen L Segota I Xian Y Tinoco R Feng Y. Prebiotic-induced anti-tumor immunity attenuates tumor growth. Cell Rep (2020) 30(6):1753–66 e6. doi: 10.1016/j.celrep.2020.01.035 32049008 PMC7053418

[71] Arpaia N Campbell C Fan X Dikiy S van der Veeken J deRoos P. Metabolites produced by commensal bacteria promote peripheral regulatory T-cell generation. Nature (2013) 504(7480):451–5. doi: 10.1038/nature12726 PMC3869884 24226773

[72] Trompette A Gollwitzer ES Yadava K Sichelstiel AK Sprenger N Ngom-Bru C. Gut microbiota metabolism of dietary fiber influences allergic airway disease and hematopoiesis. Nat Med (2014) 20(2):159–66. doi: 10.1038/nm.3444 24390308

[73] Bartolomaeus H Balogh A Yakoub M HOmann S Marko L Hoges S. Short-chain fatty acid propionate protects from hypertensive cardiovascular damage. Circulation (2019) 139(11):1407–21. doi: 10.1161/CIRCULATIONAHA.118.036652 PMC6416008 30586752

[74] Shah RR. Safety and tolerability of histone deacetylase (Hdac) inhibitors in oncology. Drug Saf (2019) 42(2):235–45. doi: 10.1007/s40264-018-0773-9 30649740

[75] Cao M Zhang Z Han S Lu X. Butyrate inhibits the proliferation and induces the apoptosis of colorectal cancer hct116 cells via the deactivation of mtor/S6k1 signaling mediated partly by sirt1 downregulation. Mol Med Rep (2019) 19(5):3941–7. doi: 10.3892/mmr.2019.10002 30864709

[76] Lund PJ Gates LA Leboeuf M Smith SA Chau L Lopes M. Stable isotope tracing in vivo reveals a metabolic bridge linking the microbiota to host histone acetylation. Cell Rep (2022) 41(11):111809. doi: 10.1016/j.celrep.2022.111809 36516747 PMC9994635

[77] Wu H van der Pol WJ Dubois LG Morrow CD Tollefsbol TO. Dietary supplementation of inulin contributes to the prevention of estrogen receptor-negative mammary cancer by alteration of gut microbial communities and epigenetic regulations. Int J Mol Sci (2023) 24(10). doi: 10.3390/ijms24109015 PMC10218871 37240357

[78] Fernandez J Ledesma E Monte J Millan E Costa P de la Fuente VG. Traditional processed meat products re-designed towards inulin-rich functional foods reduce polyps in two colorectal cancer animal models. Sci Rep (2019) 9(1):14783. doi: 10.1038/s41598-019-51437-w 31616028 PMC6794276

[79] Casali P Shen T Xu Y Qiu Z Chupp DP Im J. Estrogen reverses hdac inhibitor-mediated repression of aicda and class-switching in antibody and autoantibody responses by downregulation of mir-26a. Front Immunol (2020) 11:491. doi: 10.3389/fimmu.2020.00491 32265934 PMC7105609

[80] Sanchez HN Moroney JB Gan H Shen T Im JL Li T. B cell-intrinsic epigenetic modulation of antibody responses by dietary fiber-derived short-chain fatty acids. Nat Commun (2020) 11(1):60. doi: 10.1038/s41467-019-13603-6 31896754 PMC6940392

[81] White CA Pone EJ Lam T Tat C Hayama KL Li G. Histone deacetylase inhibitors upregulate B cell micrornas that silence aid and blimp-1 expression for epigenetic modulation of antibody and autoantibody responses. J Immunol (2014) 193(12):5933–50. doi: 10.4049/jimmunol.1401702 PMC4258531 25392531

[82] Shen T Sanchez HN Zan H Casali P. Genome-wide analysis reveals selective modulation of micrornas and mrnas by histone deacetylase inhibitor in B cells induced to undergo class-switch DNA recombination and plasma cell differentiation. Front Immunol (2015) 6:627. doi: 10.3389/fimmu.2015.00627 26697020 PMC4677488

[83] Waldecker M Kautenburger T Daumann H Busch C Schrenk D. Inhibition of histone-deacetylase activity by short-chain fatty acids and some polyphenol metabolites formed in the colon. J Nutr Biochem (2008) 19(9):587–93. doi: 10.1016/j.jnutbio.2007.08.002 18061431

[84] Furusawa Y Obata Y Fukuda S Endo TA Nakato G Takahashi D. Commensal microbe-derived butyrate induces the differentiation of colonic regulatory T cells. Nature (2013) 504(7480):446–50. doi: 10.1038/nature12721 24226770

[85] Park J Kim M Kang SG Jannasch AH Cooper B Patterson J. Short-chain fatty acids induce both effector and regulatory T cells by suppression of histone deacetylases and regulation of the mtor-S6k pathway. Mucosal Immunol (2015) 8(1):80–93. doi: 10.1038/mi.2014.44 24917457 PMC4263689

[86] Klose CS Kiss EA Schwierzeck V Ebert K Hoyler T d’Hargues Y. A T-bet gradient controls the fate and function of ccr6-rorgammat+ Innate lymphoid cells. Nature (2013) 494(7436):261–5. doi: 10.1038/nature11813 23334414

[87] Wang S Xia P Chen Y Qu Y Xiong Z Ye B. Regulatory innate lymphoid cells control innate intestinal inflammation. Cell (2017) 171(1):201–16 e18. doi: 10.1016/j.cell.2017.07.027 28844693

[88] Sepahi A Liu Q Friesen L Kim CH. Dietary fiber metabolites regulate innate lymphoid cell responses. Mucosal Immunol (2021) 14(2):317–30. doi: 10.1038/s41385-020-0312-8 PMC7736174 32541842

[89] Artis D Spits H. The biology of innate lymphoid cells. Nature (2015) 517(7534):293–301. doi: 10.1038/nature14189 25592534

[90] Munoz M Eidenschenk C Ota N Wong K Lohmann U Kuhl AA. Interleukin-22 induces interleukin-18 expression from epithelial cells during intestinal infection. Immunity (2015) 42(2):321–31. doi: 10.1016/j.immuni.2015.01.011 25680273

[91] Emgard J Kammoun H Garcia-Cassani B Chesne J Parigi SM Jacob JM. Oxysterol sensing through the receptor gpr183 promotes the lymphoid-tissue-inducing function of innate lymphoid cells and colonic inflammation. Immunity (2018) 48(1):120–32 e8. doi: 10.1016/j.immuni.2017.11.020 29343433 PMC5772175

[92] Maizels RM. Parasitic helminth infections and the control of human allergic and autoimmune disorders. Clin Microbiol Infect (2016) 22(6):481–6. doi: 10.1016/j.cmi.2016.04.024 27172808

[93] Arifuzzaman M Won TH Li TT Yano H Digumarthi S Heras AF. Inulin fibre promotes microbiota-derived bile acids and type 2 inflammation. Nature (2022) 611(7936):578–84. doi: 10.1038/s41586-022-05380-y PMC10576985 36323778

[94] Karta MR Broide DH Doherty TA. Insights into group 2 innate lymphoid cells in human airway disease. Curr Allergy Asthma Rep (2016) 16(1):8. doi: 10.1007/s11882-015-0581-6 26746844 PMC5026503

[95] McLoughlin R Berthon BS Rogers GB Baines KJ Leong LEX Gibson PG. Soluble fibre supplementation with and without a probiotic in adults with asthma: A 7-day randomised, double blind, three way cross-over trial. EBioMedicine (2019) 46:473–85. doi: 10.1016/j.ebiom.2019.07.048 PMC6712277 31375426

[96] Lewis G Wang B Shafiei Jahani P Hurrell BP Banie H Aleman Muench GR. Dietary fiber-induced microbial short chain fatty acids suppress ilc2-dependent airway inflammation. Front Immunol (2019) 10:2051. doi: 10.3389/fimmu.2019.02051 31620118 PMC6760365

[97] Ouyang W O’Garra A. Il-10 family cytokines il-10 and il-22: from basic science to clinical translation. Immunity (2019) 50(4):871–91. doi: 10.1016/j.immuni.2019.03.020 30995504

[98] Dudakov JA Hanash AM van den Brink MR. Interleukin-22: immunobiology and pathology. Annu Rev Immunol (2015) 33:747–85. doi: 10.1146/annurev-immunol-032414-112123 PMC4407497 25706098

[99] Correa RO Castro PR Fachi JL Nirello VD El-Sahhar S Imada S. Inulin diet uncovers complex diet-microbiota-immune cell interactions remodeling the gut epithelium. Microbiome (2023) 11(1):90. doi: 10.1186/s40168-023-01520-2 37101209 PMC10131329

[100] Zou J Chassaing B Singh V Pellizzon M Ricci M Fythe MD. Fiber-mediated nourishment of gut microbiota protects against diet-induced obesity by restoring il-22-mediated colonic health. Cell Host Microbe (2018) 23(1):41–53 e4. doi: 10.1016/j.chom.2017.11.003 29276170 PMC6005180

[101] Yang W Yu T Huang X Bilotta AJ Xu L Lu Y. Intestinal microbiota-derived short-chain fatty acids regulation of immune cell il-22 production and gut immunity. Nat Commun (2020) 11(1):4457. doi: 10.1038/s41467-020-18262-6 32901017 PMC7478978

[102] Chun E Lavoie S Fonseca-Pereira D Bae S Michaud M Hoveyda HR. Metabolite-sensing receptor ffar2 regulates colonic group 3 innate lymphoid cells and gut immunity. Immunity (2019) 51(5):871–84 e6. doi: 10.1016/j.immuni.2019.09.014 31628054 PMC6901086

[103] Blasius AL Beutler B. Intracellular toll-like receptors. Immunity (2010) 32(3):305–15. doi: 10.1016/j.immuni.2010.03.012 20346772

[104] Capitan-Canadas F Ortega-Gonzalez M Guadix E Zarzuelo A Suarez MD de Medina FS. Prebiotic oligosaccharides directly modulate proinflammatory cytokine production in monocytes via activation of tlr4. Mol Nutr Food Res (2014) 58(5):1098–110. doi: 10.1002/mnfr.201300497 24549946

[105] Liu B Qian J Wang Q Wang F Ma Z Qiao Y. Butyrate protects rat liver against total hepatic ischemia reperfusion injury with bowel congestion. PloS One (2014) 9(8):e106184. doi: 10.1371/journal.pone.0106184 25171217 PMC4149529

[106] Qiao YL Qian JM Wang FR Ma ZY Wang QW. Butyrate protects liver against ischemia reperfusion injury by inhibiting nuclear factor kappa B activation in kupffer cells. J Surg Res (2014) 187(2):653–9. doi: 10.1016/j.jss.2013.08.028 24445056

[107] Luhrs H Gerke T Muller JG Melcher R Schauber J Boxberge F. Butyrate inhibits nf-kappab activation in lamina propria macrophages of patients with ulcerative colitis. Scand J Gastroenterol (2002) 37(4):458–66. doi: 10.1080/003655202317316105 11989838

[108] Cleophas MC Crisan TO Lemmers H Toenhake-Dijkstra H Fossati G Jansen TL. Suppression of monosodium urate crystal-induced cytokine production by butyrate is mediated by the inhibition of class I histone deacetylases. Ann Rheum Dis (2016) 75(3):593–600. doi: 10.1136/annrheumdis-2014-206258 25589513

[109] Zhang Q Xiao X Zheng J Li M Yu M Ping F. Maternal inulin supplementation alters hepatic DNA methylation profile and improves glucose metabolism in offspring mice. Front Physiol (2020) 11:70. doi: 10.3389/fphys.2020.00070 32116778 PMC7020697

[110] Alptekin IM Cakiroglu FP Kiremitci S Recber T Nemutlu E. Inulin may prevent steatosis by suppressing cannabinoid receptor-1 and patatin-like phospholipase-3 expression in liver. Nutrition (2022) 103-104:111742. doi: 10.1016/j.nut.2022.111742 35908495

[111] Chen B Shi Y Zhang K Chang Y Fu P Liu P. Inulin reduces liver triacylglycerol by increasing lipid droplet lipolysis in fat-loaded mice. Food Res Int (2023) 163:112226. doi: 10.1016/j.foodres.2022.112226 36596155

[112] Kumar J Rani K Datt C. Molecular link between dietary fibre, gut microbiota and health. Mol Biol Rep (2020) 47(8):6229–37. doi: 10.1007/s11033-020-05611-3 32623619

[113] Zhao L Zhang F Ding X Wu G Lam YY Wang X. Gut bacteria selectively promoted by dietary fibers alleviate type 2 diabetes. Science (2018) 359(6380):1151–6. doi: 10.1126/science.aao5774 29590046

[114] Agus A Planchais J Sokol H. Gut microbiota regulation of tryptophan metabolism in health and disease. Cell Host Microbe (2018) 23(6):716–24. doi: 10.1016/j.chom.2018.05.003 29902437

[115] Campesato LF Budhu S Tchaicha J Weng CH Gigoux M Cohen IJ. Blockade of the ahr restricts a treg-macrophage suppressive axis induced by L-kynurenine. Nat Commun (2020) 11(1):4011. doi: 10.1038/s41467-020-17750-z 32782249 PMC7419300

[116] Zelante T Iannitti RG Cunha C De Luca A Giovannini G Pieraccini G. Tryptophan catabolites from microbiota engage aryl hydrocarbon receptor and balance mucosal reactivity via interleukin-22. Immunity (2013) 39(2):372–85. doi: 10.1016/j.immuni.2013.08.003 23973224

[117] Dodd D Spitzer MH Van Treuren W Merrill BD Hryckowian AJ Higginbottom SK. A gut bacterial pathway metabolizes aromatic amino acids into nine circulating metabolites. Nature (2017) 551(7682):648–52. doi: 10.1038/nature24661 PMC5850949 29168502

[118] Lamas B Richard ML Leducq V Pham HP Michel ML Da Costa G. Card9 impacts colitis by altering gut microbiota metabolism of tryptophan into aryl hydrocarbon receptor ligands. Nat Med (2016) 22(6):598–605. doi: 10.1038/nm.4102 27158904 PMC5087285

[119] Quintana FJ Basso AS Iglesias AH Korn T Farez MF Bettelli E. Control of T(Reg) and T(H)17 cell differentiation by the aryl hydrocarbon receptor. Nature (2008) 453(7191):65–71. doi: 10.1038/nature06880 18362915

[120] Daskova N Modos I Krbcova M Kuzma M Pelantova H Hradecky J. Multi-omics signatures in new-onset diabetes predict metabolic response to dietary inulin: findings from an observational study followed by an interventional trial. Nutr Diabetes (2023) 13(1):7. doi: 10.1038/s41387-023-00235-5 37085526 PMC10121613

[121] Mitchell CM Davy BM Ponder MA McMillan RP Hughes MD Hulver MW. Prebiotic inulin supplementation and peripheral insulin sensitivity in adults at elevated risk for type 2 diabetes: A pilot randomized controlled trial. Nutrients (2021) 13(9). doi: 10.3390/nu13093235 PMC8471706 34579112

[122] Vaghef-Mehrabani E Harouni R Behrooz M Ranjbar F Asghari-Jafarabadi M Ebrahimi-Mameghani M. Effects of inulin supplementation on inflammatory biomarkers and clinical symptoms of women with obesity and depression on a calorie-restricted diet: A randomised controlled clinical trial. Br J Nutr (2023) 129(11):1897–907. doi: 10.1017/S000711452200232X 36059088

[123] Wijaya H Tjahjono Y Foe K Setiadi DA Kasih E Wihadmadyatami H. Pre-meal high-performance inulin supplementation reduce post-prandial glycaemic response in healthy subjects: A repeated single-arm clinical trial. Diabetes Metab Syndr (2022) 16(1):102354. doi: 10.1016/j.dsx.2021.102354 34920203

[124] Birkeland E Gharagozlian S Birkeland KI Holm OKS Thorsby PM Aas AM. Effect of inulin-type fructans on appetite in patients with type 2 diabetes: A randomised controlled crossover trial. J Nutr Sci (2021) 10:e72. doi: 10.1017/jns.2021.70 34589204 PMC8453458

[125] Roshanravan N Alamdari NM Jafarabadi MA Mohammadi A Shabestari BR Nasirzadeh N. Effects of oral butyrate and inulin supplementation on inflammation-induced pyroptosis pathway in type 2 diabetes: A randomized, double-blind, placebo-controlled trial. Cytokine (2020) 131:155101. doi: 10.1016/j.cyto.2020.155101 32315958

[126] Cai X Yu H Liu L Lu T Li J Ji Y. Milk powder co-supplemented with inulin and resistant dextrin improves glycemic control and insulin resistance in elderly type 2 diabetes mellitus: A 12-week randomized, double-blind, placebo-controlled trial. Mol Nutr Food Res (2018) 62(24):e1800865. doi: 10.1002/mnfr.201800865 30346655

[127] Companys J Calderon-Perez L Pla-Paga L Llaurado E Sandoval-Ramirez BA Gosalbes MJ. Effects of enriched seafood sticks (Heat-inactivated B. AniMalis subsp. Lactis cect 8145, inulin, omega-3) on cardiometabolic risk factors and gut microbiota in abdominally obese subjects: randomized controlled trial. Eur J Nutr (2022) 61(7):3597–611. doi: 10.1007/s00394-022-02904-0 PMC9464132 35643872

[128] Rodriguez J Neyrinck AM Van Kerckhoven M GianFrancesco MA Renguet E Bertrand L. Physical activity enhances the improvement of body mass index and metabolism by inulin: A multicenter randomized placebo-controlled trial performed in obese individuals. BMC Med (2022) 20(1):110. doi: 10.1186/s12916-022-02299-z 35351144 PMC8966292

[129] He S Xiong Q Tian C Li L Zhao J Lin X. Inulin-type prebiotics reduce serum uric acid levels via gut microbiota modulation: A randomized, controlled crossover trial in peritoneal dialysis patients. Eur J Nutr (2022) 61(2):665–77. doi: 10.1007/s00394-021-02669-y 34491388

[130] Xiong Q Li L Xiao Y He S Zhao J Lin X. The effect of inulin-type fructans on plasma trimethylamine N-oxide levels in peritoneal dialysis patients: A randomized crossover trial. Mol Nutr Food Res (2023) 67(9):e2200531. doi: 10.1002/mnfr.202200531 36855809

[131] Maki KC Palacios OM Koecher K Sawicki CM Livingston KA Bell M. The relationship between whole grain intake and body weight: results of meta-analyses of observational studies and randomized controlled trials. Nutrients (2019) 11(6). doi: 10.3390/nu11061245 PMC6627338 31159235

[132] Pol K Christensen R Bartels EM Raben A Tetens I Kristensen M. Whole grain and body weight changes in apparently healthy adults: A systematic review and meta-analysis of randomized controlled studies. Am J Clin Nutr (2013) 98(4):872–84. doi: 10.3945/ajcn.113.064659 23945718

[133] Daskova N Heczkova M Modos I Hradecky J Hudcovic T Kuzma M. Protective effect of vegan microbiota on liver steatosis is conveyed by dietary fiber: implications for fecal microbiota transfer therapy. Nutrients (2023) 15(2). doi: 10.3390/nu15020454 PMC9867259 36678325

[134] Siljander H Honkanen J Knip M. Microbiome and type 1 diabetes. EBioMedicine (2019) 46:512–21. doi: 10.1016/j.ebiom.2019.06.031 PMC6710855 31257149

[135] Martel J Chang SH Ko YF Hwang TL Young JD Ojcius DM. Gut barrier disruption and chronic disease. Trends Endocrinol Metab (2022) 33(4):247–65. doi: 10.1016/j.tem.2022.01.002 35151560

[136] Lo Conte M Antonini Cencicchio M Ulaszewska M Nobili A Cosorich I Ferrarese R. A diet enriched in omega-3 pufa and inulin prevents type 1 diabetes by restoring gut barrier integrity and immune homeostasis in nod mice. Front Immunol (2022) 13:1089987. doi: 10.3389/fimmu.2022.1089987 36713378 PMC9880528

[137] Zou J Reddivari L Shi Z Li S Wang Y Bretin A. Inulin fermentable fiber ameliorates type I diabetes via il22 and short-chain fatty acids in experimental models. Cell Mol Gastroenterol Hepatol (2021) 12(3):983–1000. doi: 10.1016/j.jcmgh.2021.04.014 33940221 PMC8346662

[138] Rooks MG Garrett WS. Gut microbiota, metabolites and host immunity. Nat Rev Immunol (2016) 16(6):341–52. doi: 10.1038/nri.2016.42 PMC5541232 27231050

[139] Igarashi M Morimoto M Suto A Nakatani A Hayakawa T Hara K. Synthetic dietary inulin, fuji ff, delays development of diet-induced obesity by improving gut microbiota profiles and increasing short-chain fatty acid production. PeerJ (2020) 8:e8893. doi: 10.7717/peerj.8893 32296608 PMC7150546

[140] Guo L Xiao P Zhang X Yang Y Yang M Wang T. Inulin ameliorates schizophrenia via modulation of the gut microbiota and anti-inflammation in mice. Food Funct (2021) 12(3):1156–75. doi: 10.1039/d0fo02778b 33432310

[141] Perry RJ Peng L Barry NA Cline GW Zhang D Cardone RL. Acetate mediates a microbiome-brain-beta-cell axis to promote metabolic syndrome. Nature (2016) 534(7606):213–7. doi: 10.1038/nature18309 PMC4922538 27279214

[142] Yusuf K Saha S Umar S. Health benefits of dietary fiber for the management of inflammatory bowel disease. Biomedicines (2022) 10(6). doi: 10.3390/biomedicines10061242 PMC9220141 35740264

[143] Casellas F Borruel N Torrejon A Varela E Antolin M Guarner F. Oral oligofructose-enriched inulin supplementation in acute ulcerative colitis is well tolerated and associated with lowered faecal calprotectin. Aliment Pharmacol Ther (2007) 25(9):1061–7. doi: 10.1111/j.1365-2036.2007.03288.x 17439507

[144] Sun Q Arif M Chi Z Li G Liu CG. Macrophages-targeting mannosylated nanoparticles based on inulin for the treatment of inflammatory bowel disease (Ibd). Int J Biol Macromol (2021) 169:206–15. doi: 10.1016/j.ijbiomac.2020.12.094 33340633

[145] Shahdadi Sardou H Akhgari A Mohammadpour AH KaMali H Jafarian AH Afrasiabi Garekani H. Application of inulin/eudragit rs in 5-asa pellet coating with tuned, sustained-release feature in an animal model of ulcerative colitis. Int J Pharm (2021) 597:120347. doi: 10.1016/j.ijpharm.2021.120347 33545282

[146] Liu Z Liu F Wang W Sun C Gao D Ma J. Study of the alleviation effects of a combination of lactobacillus rhamnosus and inulin on mice with colitis. Food Funct (2020) 11(5):3823–37. doi: 10.1039/c9fo02992c 32329478

[147] Qiao H Zhao T Yin J Zhang Y Ran H Chen S. Structural characteristics of inulin and microcrystalline cellulose and their effect on ameliorating colitis and altering colonic microbiota in dextran sodium sulfate-induced colitic mice. ACS Omega (2022) 7(13):10921–32. doi: 10.1021/acsomega.1c06552 PMC8991927 35415348

[148] Del Fabbro S Calder PC Childs CE. Microbiota-independent immunological effects of non-digestible oligosaccharides in the context of inflammatory bowel diseases. Proc Nutr Soc (2020), 1–11. doi: 10.1017/S0029665120006953 32345388

[149] Akram W Garud N Joshi R. Role of inulin as prebiotics on inflammatory bowel disease. Drug Discovery Ther (2019) 13(1):1–8. doi: 10.5582/ddt.2019.01000 30880316

[150] Armstrong HK Bording-Jorgensen M Santer DM Zhang Z Valcheva R Rieger AM. Unfermented beta-fructan fibers fuel inflammation in select inflammatory bowel disease patients. Gastroenterology (2023) 164(2):228–40. doi: 10.1053/j.gastro.2022.09.034 36183751

[151] Le A Selle A Aubert P Durand T Brosseau C Bordron P. Maternal prebiotic supplementation impacts colitis development in offspring mice. Front Nutr (2022) 9:988529. doi: 10.3389/fnut.2022.988529 36687706 PMC9849907

[152] Bretin A Zou J San Yeoh B Ngo VL Winer S Winer DA. Psyllium fiber protects against colitis via activation of bile acid sensor farnesoid X receptor. Cell Mol Gastroenterol Hepatol (2023) 15(6):1421–42. doi: 10.1016/j.jcmgh.2023.02.007 PMC10148163 36828279

[153] Miles JP Zou J Kumar MV Pellizzon M Ulman E Ricci M. Supplementation of low- and high-fat diets with fermentable fiber exacerbates severity of dss-induced acute colitis. Inflammation Bowel Dis (2017) 23(7):1133–43. doi: 10.1097/MIB.0000000000001155 PMC5497995 28590342

[154] Zeng W Zhang Q Feng G Liu G Wu F Shen H. The effect of inulin-type fructans on the intestinal immune function of antibiotic-treated mice. Appl Microbiol Biotechnol (2022) 106(8):3265–78. doi: 10.1007/s00253-022-11896-0 35376973

[155] Gunn D Abbas Z Harris HC Major G Hoad C Gowland P. Psyllium reduces inulin-induced colonic gas production in ibs: mri and in vitro fermentation studies. Gut (2022) 71(5):919–27. doi: 10.1136/gutjnl-2021-324784 PMC8995815 34353864

[156] Vaziri ND Yuan J Norris K. Role of urea in intestinal barrier dysfunction and disruption of epithelial tight junction in chronic kidney disease. Am J Nephrol (2013) 37(1):1–6. doi: 10.1159/000345969 23258127 PMC3686571

[157] Cosola C Rocchetti MT Cupisti A Gesualdo L. Microbiota metabolites: pivotal players of cardiovascular damage in chronic kidney disease. Pharmacol Res (2018) 130:132–42. doi: 10.1016/j.phrs.2018.03.003 29518493

[158] Li L Xiong Q Zhao J Lin X He S Wu N. Inulin-type fructan intervention restricts the increase in gut microbiome-generated indole in patients with peritoneal dialysis: A randomized crossover study. Am J Clin Nutr (2020) 111(5):1087–99. doi: 10.1093/ajcn/nqz337 31942927

[159] Mitrovic M Stankovic-Popovic V Tolinacki M Golic N Sokovic Bajic S Veljovic K. The impact of synbiotic treatment on the levels of gut-derived uremic toxins, inflammation, and gut microbiome of chronic kidney disease patients-a randomized trial. J Ren Nutr (2023) 33(2):278–88. doi: 10.1053/j.jrn.2022.07.008 35995418

[160] Liu C Yuan P Wang Y Yang X Xu Y Zhang W. Effects of burdock inulin-type fructans exposure on the physiological function of healthy mice and their filial generation. J Vet Med Sci (2023) 85(4):425–33. doi: 10.1292/jvms.22-0530 PMC10139783 36843019

[161] Golzarand M Bahadoran Z MirmIran P Azizi F. Inulin intake and the incidence of cardiometabolic diseases: A prospective cohort study. Food Funct (2022) 13(20):10516–24. doi: 10.1039/d2fo00063f 36148807

[162] Stark JM Tibbitt CA Coquet JM. The metabolic requirements of th2 cell differentiation. Front Immunol (2019) 10:2318. doi: 10.3389/fimmu.2019.02318 31611881 PMC6776632

[163] Selle A Brosseau C Dijk W Duval A Bouchaud G Rousseaux A. Prebiotic supplementation during gestation induces a tolerogenic environment and a protective microbiota in offspring mitigating food allergy. Front Immunol (2021) 12:745535. doi: 10.3389/fimmu.2021.745535 35069524 PMC8769244

[164] Bouchaud G Castan L Chesne J Braza F Aubert P Neunlist M. Maternal exposure to gos/inulin mixture prevents food allergies and promotes tolerance in offspring in mice. Allergy (2016) 71(1):68–76. doi: 10.1111/all.12777 26424001

[165] Franck P Moneret-Vautrin DA Morisset M Kanny G Megret-Gabeaux ML Olivier JL. Anaphylactic reaction to inulin: first identification of specific iges to an inulin protein compound. Int Arch Allergy Immunol (2005) 136(2):155–8. doi: 10.1159/000083323 15650313

[166] Bacchetta J Villard F Vial T Dubourg L Bouvier R Kassai B. ‘Renal hypersensitivity’ to inulin and iga nephropathy. Pediatr Nephrol (2008) 23(10):1883–5. doi: 10.1007/s00467-008-0819-9 18535847

[167] Pirson F Detry B Pilette C. Occupational rhinoconjunctivitis and asthma caused by chicory and oral allergy syndrome associated with bet V 1-related protein. J Investig Allergol Clin Immunol (2009) 19(4):306–10. 19639727

[168] Wanandy T Honda-Okubo Y Davies NW Rose HE Heddle RJ Brown SGA. Pharmaceutical and preclinical evaluation of advax adjuvant as a dose-sparing strategy for ant venom immunotherapy. J Pharm BioMed Anal (2019) 172:1–8. doi: 10.1016/j.jpba.2019.04.017 31009889 PMC7127811

[169] Heddle R Smith A Woodman R Hissaria P Petrovsky N. Randomized controlled trial demonstrating the benefits of delta inulin adjuvanted immunotherapy in patients with bee venom allergy. J Allergy Clin Immunol (2019) 144(2):504–13 e16. doi: 10.1016/j.jaci.2019.03.035 31300280 PMC7112352

[170] Liu X Yang W Petrick JL Liao LM Wang W He N. Higher intake of whole grains and dietary fiber are associated with lower risk of liver cancer and chronic liver disease mortality. Nat Commun (2021) 12(1):6388. doi: 10.1038/s41467-021-26448-9 34737258 PMC8568891

[171] Kok DE Arron MNN Huibregtse T Kruyt FM Bac DJ van Halteren HK. Association of habitual preoperative dietary fiber intake with complications after colorectal cancer surgery. JAMA Surg (2021) 156(9):1–10. doi: 10.1001/jamasurg.2021.2311 PMC8209565 34132738

[172] Mauro MO Monreal MT Silva MT Pesarini JR Mantovani MS Ribeiro LR. Evaluation of the antimutagenic and anticarcinogenic effects of inulin in vivo. Genet Mol Res (2013) 12(3):2281–93. doi: 10.4238/2013.July.8.9 23884771

[173] Taper HS Roberfroid MB. Inulin/oligofructose and anticancer therapy. Br J Nutr (2002) 87 Suppl 2:S283–6. doi: 10.1079/BJNBJN/2002549 12088530

[174] Hayday AC. Gammadelta T cell update: adaptate orchestrators of immune surveillance. J Immunol (2019) 203(2):311–20. doi: 10.4049/jimmunol.1800934 31285310

[175] Ali MS Hussein RM Gaber Y Hammam OA Kandeil MA. Modulation of jnk-1/beta-catenin signaling by lactobacillus casei, inulin and their combination in 1,2-dimethylhydrazine-induced colon cancer in mice. RSC Adv (2019) 9(50):29368–83. doi: 10.1039/c9ra04388h PMC9071812 35528422

[176] Hu C Xu B Wang X Wan WH Lu J Kong D. Gut microbiota-derived short-chain fatty acids regulate group 3 innate lymphoid cells in hcc. Hepatology (2023) 77(1):48–64. doi: 10.1002/hep.32449 35262957 PMC9970019

[177] Oliero M Hajjar R Cuisiniere T Fragoso G Calve A Santos MM. Inulin impacts tumorigenesis promotion by colibactin-producing escherichia coli in apc(Min/+) mice. Front Microbiol (2023) 14:1067505. doi: 10.3389/fmicb.2023.1067505 36819017 PMC9932902

[178] Oliero M Hajjar R Cuisiniere T Fragoso G Calve A Dagbert F. Prevalence of pks + Bacteria and enterotoxigenic bacteroides fragilis in patients with colorectal cancer. Gut Pathog (2022) 14(1):51. doi: 10.1186/s13099-022-00523-y 36578036 PMC9798702

[179] Singh V Yeoh BS Chassaing B Xiao X Saha P Aguilera Olvera R. Dysregulated microbial fermentation of soluble fiber induces cholestatic liver cancer. Cell (2018) 175(3):679–94 e22. doi: 10.1016/j.cell.2018.09.004 30340040 PMC6232850

